# Tailoring the pore size of expanded porphyrinoids for lanthanide selectivity†

**DOI:** 10.1039/d3ra05710k

**Published:** 2023-09-27

**Authors:** Thomas Malcomson, Lewis Edwards-Yates, Andrew Kerridge

**Affiliations:** a Department of Chemistry, School of Natural Sciences, University of Manchester Oxford Road Manchester M13 9PL UK thomas.malcomson@manchester.ac.uk; b Faculty of Chemistry & Earth Sciences, Priestley College UK; c Department of Chemistry, Lancaster University UK a.kerridge@lancaster.ac.uk

## Abstract

Despite increase in demand, capacity for the recycling of rare earth elements remains limited, partly due to the inefficiencies with processes currently utilised in the separation of lanthanides. This study highlights the potential use of expanded porphyrinoids in lanthanide separation through selective binding, dependent on the tailored pore size of the macrocycle. Each emerging trend is subjected to multi-factored analysis to decompose the underlying source. Results promote the viability of size-based separation with preferential binding of larger lanthanum(iii) ions to amethyrin and isoamethyrin macrocycles, while smaller macrocycles such as pentaphyrin(0.0.0.0.0) present a preferential binding of lutetium(iii) ions. Additionally, the porphyrin(2.2.2.2) macrocycle shows a selectivity for gadolinium(iii) ions over both larger and smaller ions. An upper limit of applicable pore size is shown to be ≈2.8 Å, beyond which the formed complexes are predicted to be less stable than the corresponding nitrate complexes.

## Abstract 2

Selective tailoring of the pore in expanded porphyrinoids is presented as a novel approach for the separation of lanthanide ions.

## Graphical abstract

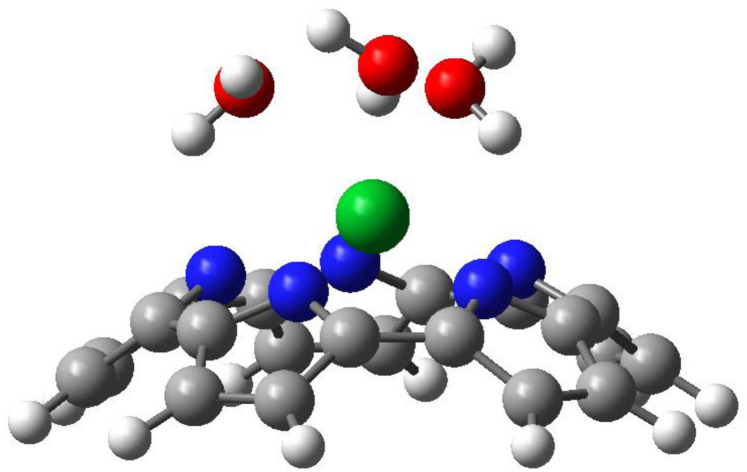

## 1 Introduction

The lanthanides (elements 57–71: La–Lu) have shown a remarkable increase in demand with developments in modern technology due to their diversity of applications ranging from medicine to nuclear fuel.^1–7^ Consequently, the lanthanides are in high demand as a resource;^8–11^ in particular, a study by the US Department of Energy in 2011 identified the lanthanides Nd, Eu, Tb, and Dy as being critical in terms of their supply risk and their importance to clean energy.^12^ Subsequent studies^13,14^ quantified the number of remaining years until depletion of lanthanide reserves under current mining operations, highlighting a desperate need for improvements in the limited recycling reported from 1994 to 2019.^15^ In addition, most of this recycling was from magnet scrap, although from 2015 small quantities have been recycled from batteries and fluorescent lamps.^16–19^ However in 2011, 45% of consumed rare earth materials were left in landfill.^20^

Over 90% of rare earth metals (REMs) are found in igneous deposits of bastnasite (70%) and monazite (20%)^21^ but they are often found with radioactive actinides such as uranium and thorium^22^ and so they must first be separated from these actinides following mining before the REMs can be separated from each other. REMs are abundant in the Earth's crust^23^ and are also produced in nuclear fission. With their array of applications, poor recycling and problematic separations, the REMs are depleting as a resource. In particular, Schuler *et al.* have identified serious concerns with the demands of La, Pr, Nd, Eu, Tb, Dy and Yb.^12^ While for many lanthanides the quantity available is sufficient in the long-term, current mining strategies fail to meet current demands,^13,15^ leading to the year-on-year increase in demand of lanthanides; hence, recent emphasis is on the recycling of lanthanides, which is hindered by the difficulty of their separations.^24–27^

The chemistry of the lanthanides is dominated by the trivalent oxidation state (Ln(iii)); across the series these cations differ primarily in their 4f orbital occupations. Considering the “core-like” nature of the 4f orbitals, the interaction between the 4f electrons and lanthanide coordination sphere, outside of electrostatics, is minimal. This property results in similar chemical behaviour being observed across the series; in response, the most viable means of conducting lanthanide separation aim to take advantage of the difference in ion size or magnetic properties; for example, recent work by Higgins *et al.* has exploited magnetism to separate lanthanides utilising magnetomigrations.^28^ Although significant advances have been made in the development and application of electrochemical recovery methods to REMs^29–34^ many of these focus on separating lanthanides and actinides, rather than separation of lanthanides from each other. To this end, this study will focus on a size-based selectivity method, utilising extended porphyrinoid macrocycles with tuneable pore sizes for separation of lanthanide ions, in particular for lanthanum (La), gadolinium (Gd) and lutetium (Lu).

Current industrial lanthanide separations are performed almost exclusively by solvent extraction which can achieve up to 99.9999% purity.^21,35^ Unfortunately, the process requires the lanthanides to be repeatedly pumped back into the system and requires a variety of different ligands to achieve any particular lanthanide-pair separation (for example Eu/Gd); with some pairs being substantially more difficult, or even unattainable, with this method. In some cases, one can appeal to the alternative oxidation states of the lanthanides such as Ce^4+^ and Eu^2+^, but in general it is extremely difficult to achieve separation of adjacent lanthanides.^36,37^

**Fig. 1:**
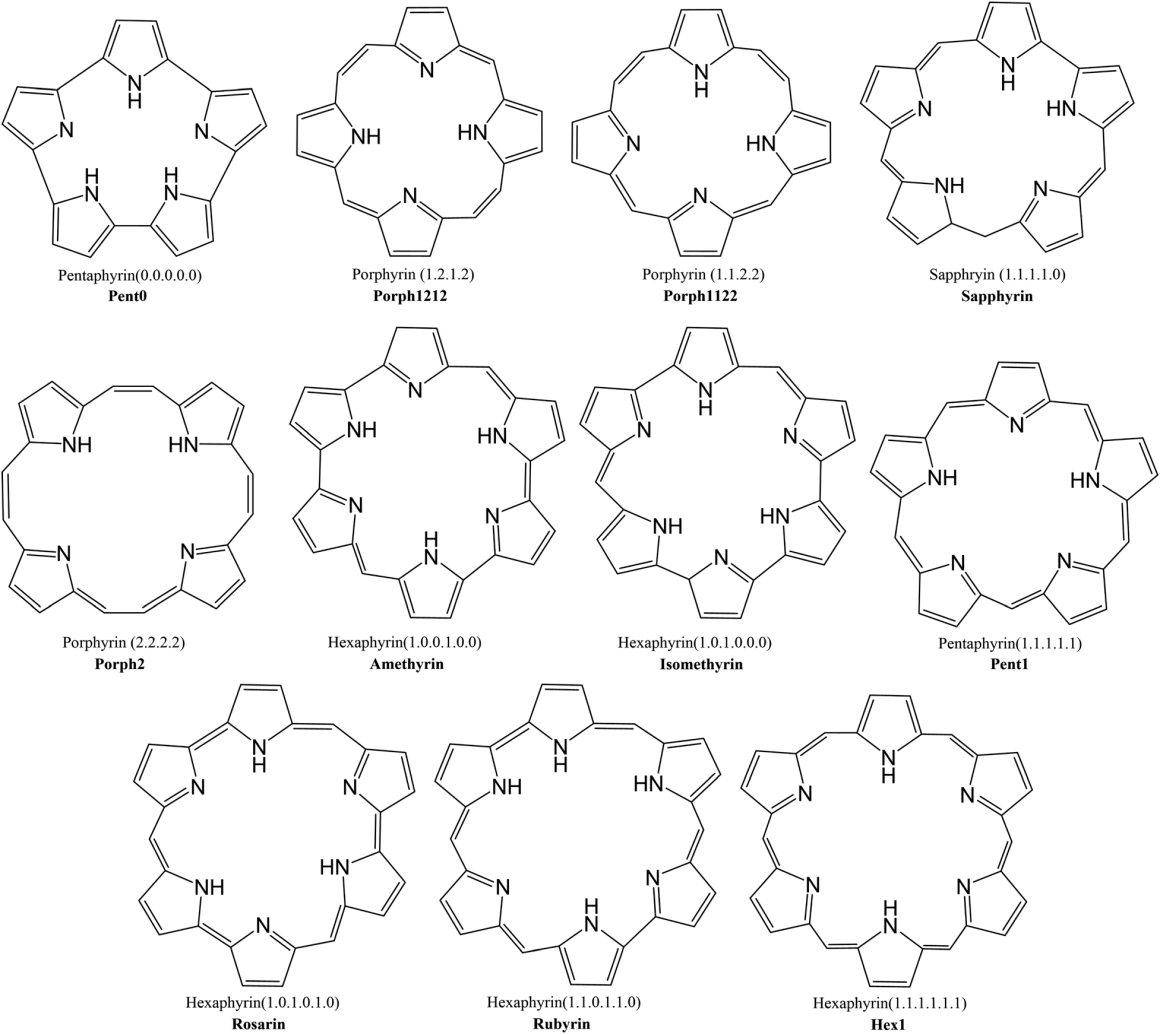
Schematic of expanded porphyrinoid macrocycles studied throughout this work. Nomenclature of (*n*.*n*.*n*.⋯.*n*), where *n* = 0, 1, 2, is used to denote the number of –CH– groups bridging each pyrrole group within a given macrocycle.

This investigation examines the potential ability of the expanded porphyrinoids (EPs) with varying core sizes to probe the potential of these ligands for lanthanide separation. Porphyrin is a tetrapyrrolic macrocycle and occurs in the prosthetic group of haemoglobin, referred to as haem, where it coordinates iron(ii). Fe^2+^ has 24 electrons, compared with the 54 to 68 of the trivalent lanthanides, and so larger ligands are needed to accommodate the size of the lanthanide: by increasing the number of pyrrole rings (from 4 to 6) and by varying the number of bridging carbons between these pyrrole rings (from 0 to 2), one can devise a collection of size-varying pentaphyrins and hexaphyrins (Fig. 1) to complex with the lanthanides. Synthetically, each of these EPs is well understood and have been shown to be readily produced and easily modifiable,^38,39^ with diverse synthetic routes proposed for many of the macrocycles shown in Fig. 1,^40–47^ and many already presenting as promising targets in hydrogen storage and photodynamic therapy.

## 2 Methods

Macrocycles presented in Fig. 1 were selected to provide wide range of pore sizes (Fig. 3) while also incorporating a range of pyrrolic groups and overall macrocycle flexibility. The selected lanthanide ions (La(iii), Gd(iii), and Lu(iii)) were chosen for two reasons: firstly, representing a significant range in ion size allows for the best intuitive look at the relationship between pore size and ion selectivity; secondly, these ions possess a valence configuration of 4f^0^, 4f^7^, and 4f^14^, respectively, resulting in complexes that can be expected to be monodeterminantal and, as such, suitable for description with density functional theory.

Geometric structures were optimised using the BHLYP/def2-SVP model chemistry^48–53^ utilising a small-core ECP on each lanthanide centre;^54^ verification of structural minima were conducted through frequency analysis. Structural minima were additionally verified with the def2-TZVP basis set^53^ and energies quoted throughout this work where determined utilising single-point energy calculations with the BHLYP/def2-TZVP model chemistry at the BHLYP/def2-SVP geometry. The conductor-like screening model (COSMO)^55^ was also incorporated into evaluating single point energies. Spin–orbit coupling was neglected in these calculations under the assumption that any spin–orbit coupling component present would be expected to be of a similar magnitude in both the nitrate and EP complexes and should therefore largely cancel in the exchange reactions. We have previously applied this approximation in recent studies of f-element complexation by both BTP and BTPhen ligands.^56,57^

Each of the macrocycles were assumed to be fully deprotonated when bound to the Ln(iii) ion, resulting in 4-membered macrocycles with a −2 charge which was then balanced with the inclusion of a nitrate ion for a total of −3 to correspond to the +3 of the ion. Larger ring systems were assumed to adopt a −3 state, so no nitrate was included in the complex. Example La(iii) complexes can be seen in Fig. 4.

All calculations were carried out with the Turbomole 7.1 program.^58^ Structures and surfaces were visualised through the GaussView5 program.^59^

### 2.1 Quantifying macrocycle pore size

**Fig. 2:**
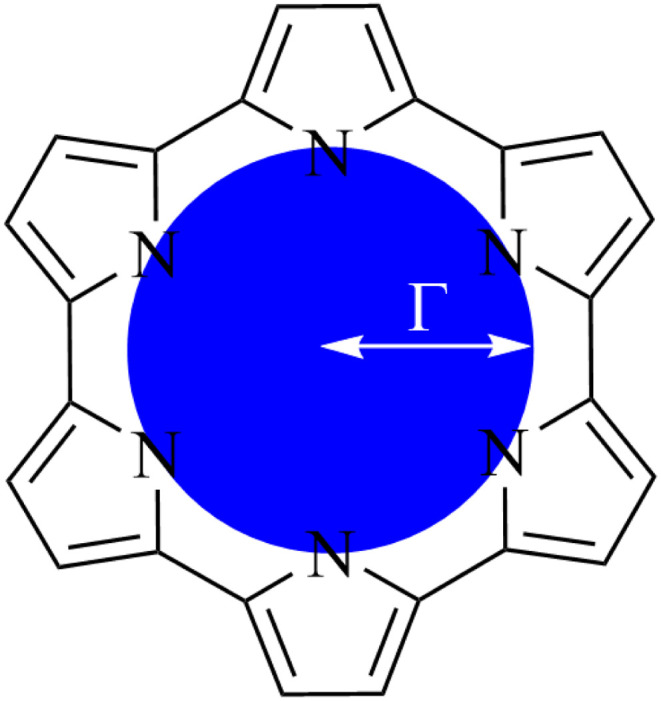
Schematic representation of circular approximation to the core size of an expanded porphyrin.

**Fig. 3:**
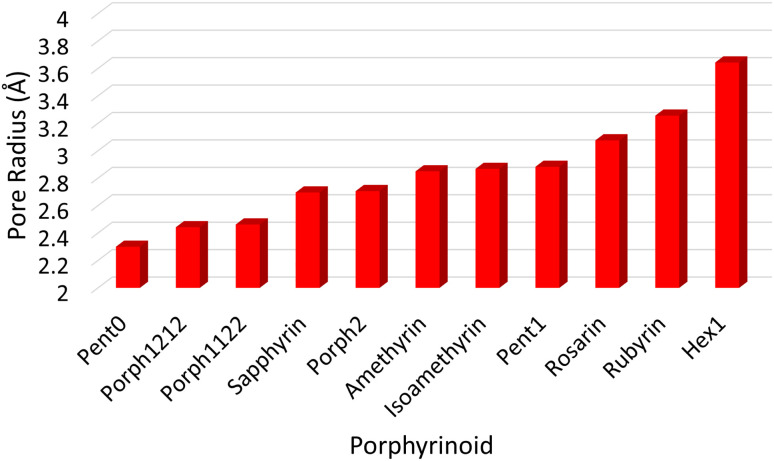
Pore sizes of selected porphyrinoids as determined by a circular approximation.

To quantify the sizes of these macrocycles, a polygonal approximation was applied and the corresponding circumradius, *Γ* (Fig. 2), was calculated for each macrocycle such that:1
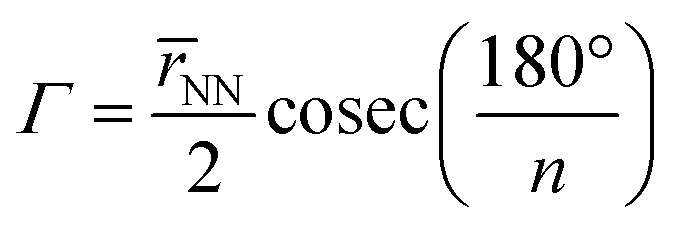
where *r̄*_NN_ is the mean distance between adjacent pyrrolic nitrogens and *n* is the number of NN distances utilised; this is the radius of the unique circle intersecting all the pyrrolic nitrogen atoms in the macrocycle, under the assumption that the N–N distances are the same and all the nitrogen atoms are coplanar. The circumradii of each macrocycle, hereafter taken to represent the pore size of the macrocycle, is presented in Fig. 3. It is worth noting that, while this assumption holds in the majority of cases, there are exceptions such as Hex1 where significant deviation from coplanarity is observed; a likely cause for this deviation is due to the increase macrocycle size and flexibility compared to other structures presented.

### 2.2 Spin-contamination

During calculations involving open-shell systems, such as those containing Gd(iii), the presence and degree of spin-contamination was also accounted for. Quantifying the extent of spin-contamination is done by considering the expectation value of the spin operator, denoted by 〈*Ŝ*^2^〉, the exact value of which is given by:^60^2
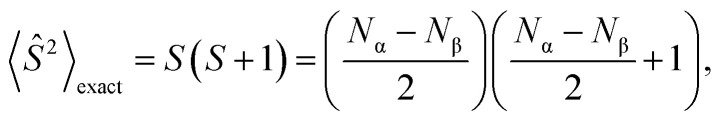
where *N*_α_ and *N*_β_ are the number of α-spin and β-spin electrons, respectively, and *S* is the total spin, resulting in 〈*Ŝ*^2^〉_exact_ = 15.75. In general for an unrestricted system, the expected spin value is:^60^3
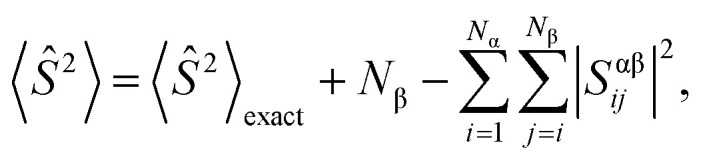
where4
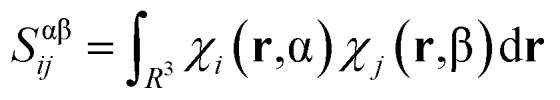
is the spatial overlap integral between α-orbital *i* and β-orbital *j*; these are non-zero by the construction of the unrestricted determinant (*i.e.* the spatial orbitals are non-orthogonal). For the gadolinium nitrate 〈*Ŝ*^2^〉 = 15.76, which shows good agreement with the theoretical value. In contrast, spin-contamination within the Gd(iii)–EP complexes showed higher sensitivity to the basis set and the incorporation of the solvent model (COSMO). However, use of the BHLYP/TZVP_COSMO_ model chemistry was found to reduce the spin-contamination to negligible values for the systems presented here, equivalent to those of the nitrate complex.

### 2.3 Exchange reactions

Selectivity was determined through the consideration of a series of exchange reactions between pairs of Ln(iii) ions such that:

5 Ln(1)(EP)(NO_3_)_*x*_(H_2_O)_*y*_ + Ln(2)(NO_3_)_3_(H_2_O)_*n*_ ⇌ Ln(2)(EP)(NO_3_)_*x*_(H_2_O)_*y*_ + Ln(1)(NO_3_)_3_(H_2_O)_*n*_

where the first component is in the form of a charge neutral Ln(EP)(NO_2_)_*x*_(H_2_O)_*y*_ complex where *x* = 0 or 1 depending on the charge of a given EP; the second ion in the exchange is considered in the form of a charge neutral Ln(NO_2_)_3_(H_2_O)_*n*_ complex where *n* = 2 or 3 depending on the preference of a given Ln(iii) ion for an 8- or 9-coordinate ligand sphere. It is worth noting that, for the extent of this study, while Gd(iii) is known to capable of adopting either an 8- or 9-coordinate ligand sphere, we have assumed that, within both the nitrate and EP complexes, Gd(iii) adopts a 8-coordinate ligand sphere.

Reaction energies (Δ*G*^TZVP+COSMO^_exchange_) presented throughout this work have been constructed by applying the free energy correction from the BHLYP/SVP model chemistry to the SCF energy provided by the BHLYP/TZVP_COSMO_ single-point calculation due to the large system size and, as such, large cost involved with determining the free energy correction at the larger model chemistry.

## 3 Results & discussion

### 3.1 Ligand pore size

The pore size, modelled by the circumradius (*Γ*) (Fig. 2), of each EP studied was shown to form a reliably consistent trend (Fig. 3) ranging from 2.2 Å (Pent0) to 3.6 Å (Hex1). While the trend in pore size cannot be considered purely as a function of the number of pyrrolic groups present in a given macrocycle, it is observed that the trend is instead defined by the number of total atoms comprising the internal ring of the structure, with the presence of bridging carbons leading to a reduced pore size compared to more rigid structures containing a similar number of atoms.

**Fig. 4:**
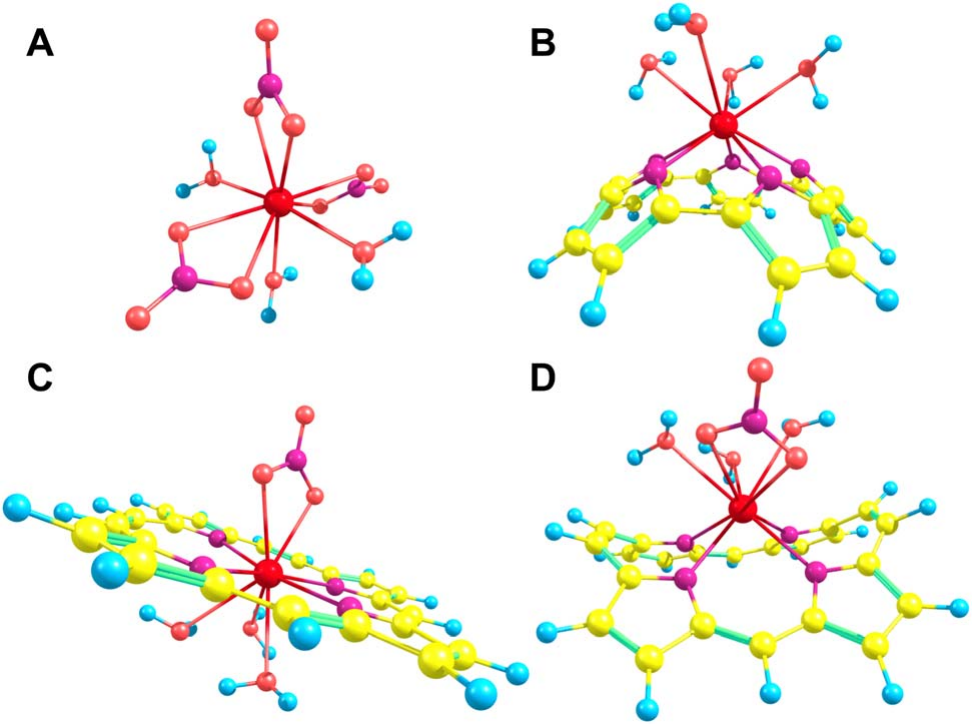
Selection of La(iii) complexes utilised throughout this study: (A) La(NO_3_)_3_(H_2_O)_3_; (B) La(Pent0)(H_2_O)_4_; (C) La(Porph1122)(H_2_O)_3_NO_3_; (D) La(Porph1212)NO_3_(H_2_O)_3_. Yellow – carbon; blue – hydrogen; pale red – oxygen; purple – nitrogen; bright red – lanthanum.

**Fig. 5:**
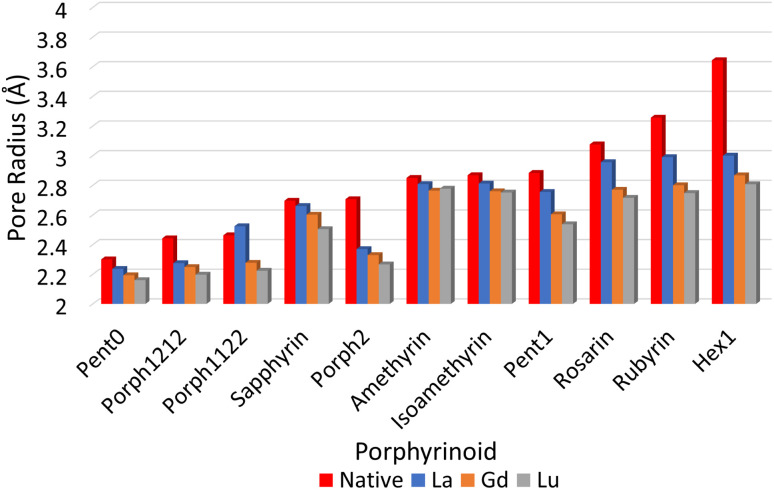
Pore sizes of selected EP macrocycles both at their respective geometric minima (red), and complexed to each Ln(iii) ion, as determined by a circular approximation, as in eqn (1).

Upon binding to a Ln(iii) ion (Fig. 5), the prevalence of this trend is no longer upheld with the resulting, distorted, pore sizes showing a significant degree of variation throughout the series. However, with the exception of the La complex of Porph1122, complexation of the EP and Ln(iii) ion results in a reduction in overall pore size for each structure studied; and, with the exception of the Gd complex of Amethyrin, the distorted pore size is shown to decrease with increasing ion size (La → Lu). The outlying increase in pore size observed in the La-Porph1122 is explained by macrocycle stretching to accommodate coordination by water molecules above the plane and the nitrate ion below the plane (Fig. 4C); this is in comparison to the equivalent La-Porph1212 complex (Fig. 4D) where the trans conformation of the –C_2_H_2_– and –CH– linkers allows the complex to accommodate the bowed structure observed in order to facilitate all solvent ligands to coordinate on one side of the complex.

Notable structures within the series are the Amethryin and Isoamethyrin pair which show minimal distortion upon ion complexation. This lack of distortion in pore size can be considered to primarily be a result of the lack of bridging carbons resulting in reduced flexibility in the ring, further explaining the small variation observed in the Pent0 complexes. Conversely, Porph2 and Hex1 show the largest reduction in pore size; for Porph2, the presence of two bridging carbons between each pyrrolic ring results in a significantly higher degree of flexibility which allows for large changes in pore size without a comparatively high reorganisation energy within the EP (Fig. 7). However, while the Hex1 structure contains a reasonable degree of flexibility, the primary factor driving the large degree of contraction in pore size is that the size of the pore in the uncoordinated EP is substantially larger than can be accommodated in the coordination sphere of any of the Ln(iii) ions. As a result, significant geometric distortion of the EP is required in order to incorporate complexation. This rationalisation is further supported by considering the series from Rosarin → Hex1; despite a steadily increasing pore size observed in the native structures (Fig. 5; red), there is minimal change observed in the pore size of the coordinated complexes, independent of the macrocycle. These observations suggest that, provided the macrocycle possesses sufficient flexibility, the pore sizes found in the coordinated Rosarin complexes represent the largest coordination sphere accommodated by each Ln(iii) ion. From this, a reasonable deduction could be made that application of macrocycles with a larger pore size or reduced flexibility, when compared to Rosarin, would yield less favourable results in terms of both selectivity and stability of the resulting complex, with little merit in further exploration.

### 3.2 Exchange reactions

**Fig. 6:**
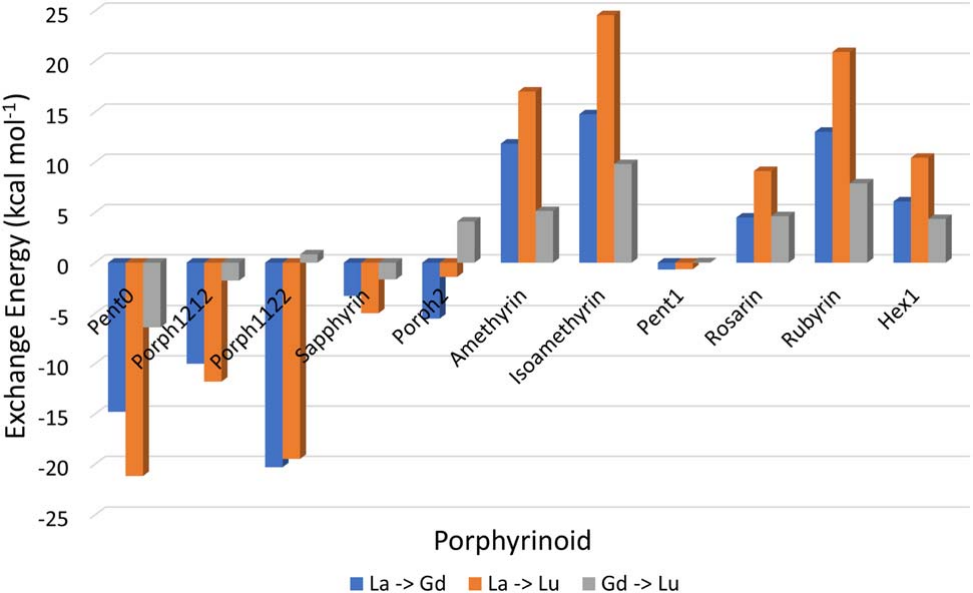
Energy changes (kcal mol^−1^) for the exchange reactions shown in eqn (5) in which the smaller ion replaces the larger ion within the EP macrocycle. As such, negative reaction energies denote a preference for EP complexation with the smaller lanthanide, while positive reaction energies denote selectivity preference for EP complexation of the larger ion.

Analysis of exchange reactions (Fig. 6) presents a trend that can be broken into three distinct regions: the first region is occupied by Pent0 → Sapphyrin; the second region is comprised of the Amethyrin and Isoamethyrin pair; and the third range encompassing Rosarin → Hex1.

The first region, comprised of rings with smaller pore sizes (Fig. 3), shows a selectivity preference for both lutetium and gadolinium over lanthanum and for lutetium over gadolinium (Fig. 6). With the exception of Porph1122, this preference for a smaller ion is also observed across this region of the series (Fig. 6; grey) with Porph1122 instead showing negligible preference for either gadolinium or lutetium.

The second region represents an inversion in selection preference for larger ions; with lanthanum bound structures seeming more stable than those containing either gadolinium or lutetium and with lutetium selected against in all exchange reactions. It is worth noting that the asymmetric Isoamethyrin presents a greater selectivity preference than the symmetric Amethyrin structure.

The first and second regions are separated by Porph2 which, while maintaining a preference for lutetium over lanthanum, shows a preference for gadolinium. Porph2 therefore represents an inflection point between the two regions while also highlighting the potential to select for central lanthanides in addition to selecting for the extremes.

Pent1, which sits between the second and third region, shows negligible selection preference across the three ions studied, followed by a region again showing an energetic preference towards lanthanum over either of the smaller ions. Within this third region Rubyrin is shown to exhibit a significantly greater selectivity than either Rosarin or Hex1.

It is worth noting that, across the series, the relationship:

6 Δ*E*_La→Gd_ + Δ*E*_Gd→Lu_ = Δ*E*_La→Lu_

predominantly holds across the series, suggesting that the electrostatic interactions between the EP and a given ion is equivalent across the lanthanide series, leaving the dominant factors in determination of selectivity dependent upon optimal pairing of the Ln(iii) ion with a given pore size, and the steric strain required to distort a given ligand from its non-coordinated geometry.

### 3.3 Distortion energies

**Fig. 7:**
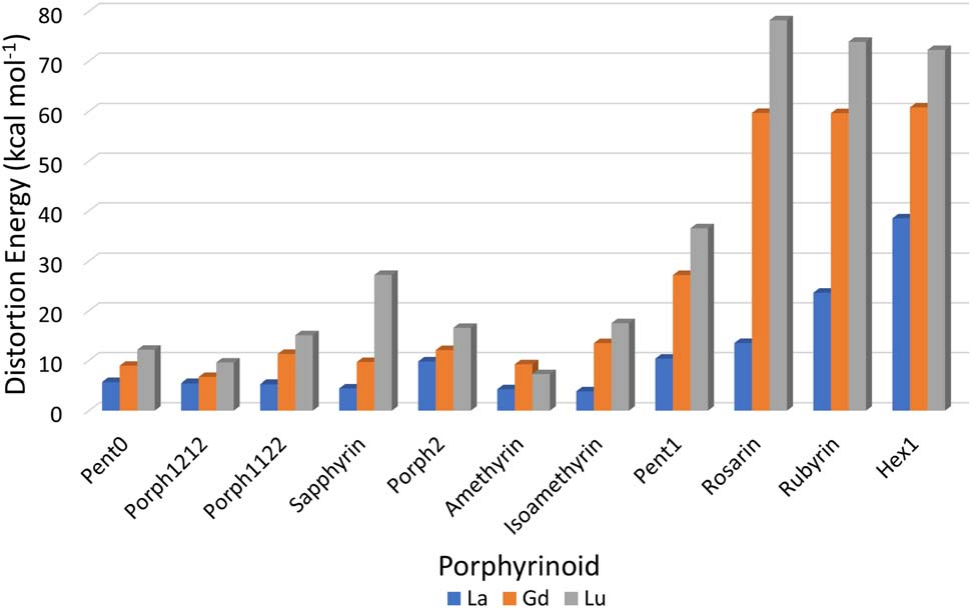
Energy differences (kcal mol^−1^) required to distort each EP macrocycle from its geometric minima to the geometry achieved when complexing a given Ln(iii) ion.

Consideration of the EP distortion energies (Fig. 7) sheds additional light into the various regions observed in the exchange reaction series (Fig. 6). Primarily, there are two observable trends in these energies: firstly, with the exception of Amethyrin, the distortion energy for any given EP increases with decreasing ion size; the second is, again with the exception of Amethyrin, the distortion energy broadly increases with increasing pore size.

While Fig. 6 suggests that the performance of Pent1, showing no preference across the series, is anomalous in relation to the overall trend, consideration of the pore size deviations (Fig. 5) and distortion energies (Fig. 7) suggest instead that, rather than signifying a case where binding of any Ln ion is equally favoured, it acts to represent a point after which binding of any lanthanide may be disfavoured compared to the nitrate complex. This inference becomes apparent through the significant, and continuous, increase in pore size distortion observed for Pent0 – Hex1 (Fig. 5) compared to other EPs. Although a similar deviation is observed for both Porph1212 and Porph2, analysis of the distortion energies (Fig. 7) shows that the geometry changes needed to facilitate the large deviations in pore size require significantly less energy than that needed for the larger EPs; this is readily accounted for by the extended length of the link-groups between each pyrrole ring providing a substantial increase in macrocycle flexibility.

## 4 Conclusions

Throughout this work we have demonstrated, and deconstructed the relationship between pore size and selectivity of lanthanide ions within a series of EP compounds.

Analysis of exchange reactions (Fig. 6) depicts a distinct switch in selection preference from the smaller lutetium, to the larger lanthanum ion with an inflection point represented by Porph2 showing potential for selectivity of central lanthanides. Table 1, summarising the selection preferences across the series, highlights that, based on exchange reaction energies alone, the tuning of pore size within the EP may be a promising route in the development of novel EPs for lanthanide separations.

**Table 1:** Selectivity orders of each expanded porphyrinoid based on the relevant energies of their exchange reactions

EP	Selection preference
Pent0	Lu > Gd > La
Porph1212	Lu > Gd > La
Porph1122	Gd > Lu > La
Sapphyrin	Lu > Gd > La
Porph2	Gd > Lu > La
Amethyrin	La > Gd > Lu
Isoamethyrin	La > Gd > Lu
Pent1	Lu > Gd > La
Rosarin	La > Gd > Lu
Rubyrin	La > Gd > Lu
Hex1	La > Gd > Lu

Combining this analysis with that of the distortion energies required for a given EP to accommodate a particular Ln(iii) ion shows that the upper-bound pore size for the design of EPs for lanthanide separation can be assumed to lie between that of Isoamethyrin and Pent1, after which it is suggested that the selectivity observed in calculations (Fig. 6 & Table 1) towards the larger lanthanum ion are an artefact resulting from the larger ion being the lesser of a series of poor choices within a given EP.

The data presented throughout this work shows not only that tuning of the macrocycle pore size can, in isolation, provide a viable route in selective lanthanide separation, with potential for tailored selection of central lanthanide ions; but also show that, despite the relatively simple electronic interaction between lanthanide and macrocycle, a multi-factored analysis is required to fully deconstruct the observed trends.

Finally, structures studied throughout this work have focused solely on the construction of a two-dimensional pore within a given macrocycle. While, ideally, each coordinating macrocycle would form the equatorial plane of the lanthanide coordination sphere, with the required solvent molecules occupying axial positions (Fig. 4C), instead, even with ligand distortion, it is more common that the Ln(iii) ion binds above the plane of the macrocycle (Fig. 4B and D), reducing the overall sensitivity of the pore-size. While this pocket does act to reduce the necessary distortion energy, this reduced control in the tuning of the pore size, allows for highly variable solvent coordination geometries. With this in mind, future investigation could be readily directed into the development of extended porphyrinoid macrocycles containing either an “arm-” or “arch-“like moiety that can act as an additional coordination point, a practice prevalent throughout the literature in developing Ln-porphyrinoids for biochemical purposes.^61–65^ Incorporation of these moieties would not only facilitate the creation of an accessible, tunable three-dimensional pore, allowing for more precise tuning of the pore size in order to more finely differentiate across the lanthanide series; but would also enable the production of a more extensive porphyrinoid network, altering the macro-level material chemistry of these compounds.

## Conflicts of interest

There are no conflicts to declare.

## Supplementary Material

RA-013-D3RA05710K-s001
