# Supplementary material for: Tailoring the pore size of expanded porphyrinoids for lanthanide selectivity
Source: RSC Adv. 2023 Sep 27;13(41):28426–33. doi: 10.1039/d3ra05710k (PMC10523133; doi:10.1039/d3ra05710k)
Supplement: RA-013-D3RA05710K-s001 [file RA-013-D3RA05710K-s001.pdf]

Electronic Supplementary Material (ESI) for RSC Advances.
This journal is © The Royal Society of Chemistry 2023

Modifying Macrocycles for Lanthanide Selectivity

Thomas Malcomson,[*,†] Lewis Edwards-Yates,[‡] and Andrew Kerridge[*,¶]

†*Department of Chemistry, School of Natural Sciences, The University of Manchester, Manchester, M13 9PL, UK.*

‡*Department of Chemistry & Earth Sciences, Priestley College, Loushers Lane, Warrington, Cheshire, WA4 6RD, UK.*

¶*Department of Chemistry, Lancaster University, Lancaster, LA1 4YB, UK; E-mail: a.kerridge@lancaster.ac.uk*

E-mail: thomas.malcomson@manchester.ac.uk; a.kerridge@lancaster.ac.uk

Energetics

Table S1: Circular approximation of macrocyclic core size (Å) by approximating the circle as a regular polygon with the average N-N bond length as the sides and with the N atoms as the vertices. The circumradii (*i.e.* the radius of the unique circle intersecting the N atoms), denoted by Γ, are recorded in the table (first row) with their standard deviations (second row). [a] Ionic radii obtained from Shannon *et al.*[1]

		La	^{8}Gd	Lu
Ionic radius[a]		1.216	1.053	0.977
Pent0	2.704	2.236	2.193	2.160
	0.000	0.010	0.004	0.003
Porph1212	2.442	2.275	2.248	2.497
	0.033	0.043	0.052	0.076
Porph1122	2.463	2.524	2.277	2.222
	0.247	0.318	0.166	0.157
Sapphyrin	2.696	2.660	2.601	2.504
	0.199	0.199	0.185	0.141
Porph2	2.706	2.369	2.329	2.266
	0.001	0.194	0.240	0.253
Amethyrin	2.850	2.808	2.764	2.777
	0.056	0.118	0.061	0.047
Isoamethyrin	2.868	2.811	2.760	2.751
	0.171	0.164	0.142	0.133
Pent1	3.389	2.755	2.604	2.537
	0.000	0.033	0.055	0.059
Rosarin	3.076	2.956	2.770	2.715
	0.154	0.106	0.064	0.060
Rubyrin	3.256	2.990	2.800	2.748
	0.187	0.114	0.094	0.085
Hex1	3.643	3.000	2.867	2.807
	0.006	0.040	0.012	0.011

Table S2: Energies of the distorted and optimised geometries of each macrocycle ($Hartrees$) and the required energy change ($kcal\ mol^{-1}$) associated with the extended porphyrinoid binding of the La(III) ion.

Macrocycle	Distorted Geometry	Optimised Geometry	Energy Difference
Pent0	-1041.238942	-1041.247978	5.669535137
Porph1212	-1065.363216	-1065.371932	5.468297647
Porph1122	-1065.351715	-1065.360132	5.280496784
Sapphyrin	-1196.157757	-1196.164861	4.456969367
Porph2	-1142.689411	-1142.705027	9.796897579
Amethyrin	-1327.241462	-1327.248307	4.294762561
Isoamethyrin	-1327.239594	-1327.245783	3.882640759
Pent1	-1234.991549	-1235.008065	10.3618938
Rosarin	-1365.812742	-1365.834258	13.49889131
Rubyrin	-1404.605585	-1404.643239	23.62335171
Hex1	-1481.917453	-1481.978783	38.47719538

Table S3: Energies of the distorted and optimised geometries of each macrocycle (*Hartrees*) and the required energy change ($kcal\ mol^{-1}$) associated with the extended porphyrinoid binding of the Gd(III) ion.

Macrocycle	Distorted Geometry	Optimised Geometry	Energy Difference
Pent0	-1041.233734	-1041.247978	8.936459705
Porph1212	-1065.361289	-1065.371932	6.677081347
Porph1122	-1065.34212	-1065.360132	11.30067381
Sapphyrin	-1196.149436	-1196.164861	9.677279388
Porph2	-1142.685771	-1142.705027	12.08057361
Amethyrin	-1327.233601	-1327.248307	9.226652616
Isoamethyrin	-1327.224306	-1327.245783	13.47439136
Pent1	-1234.964758	-1235.008065	27.17010999
Rosarin	-1365.739131	-1365.834258	59.68109216
Rubyrin	-1404.548197	-1404.643239	59.62789081
Hex1	-1481.881896	-1481.978783	60.78491306

Table S4: Energies of the distorted and optimised geometries of each macrocycle (*Hartrees*) and the required energy change ($kcal\ mol^{-1}$) associated with the extended porphyrinoid binding of the Lu(III) ion.

Macrocycle	Distorted Geometry	Optimised Geometry	Energy Difference
Pent0	-1041.228613	-1041.247978	12.14950772
Porph1212	-1065.35666	-1065.371932	9.581465763
Porph1122	-1065.336119	-1065.360132	15.06510419
Sapphyrin	-1196.121517	-1196.164861	27.1927325
Porph2	-1142.678648	-1142.705027	16.54956414
Amethyrin	-1327.236768	-1327.248307	7.239551793
Isoamethyrin	-1327.217915	-1327.245783	17.48357215
Pent1	-1234.94993	-1235.008065	36.47292275
Rosarin	-1365.709596	-1365.834258	78.21037673
Rubyrin	-1404.52547	-1404.643239	73.88598886
Hex1	-1481.863645	-1481.978783	72.23550253

Table S5: Energy changes ($kcal\ mol^{-1}$) for the exchange reactions in which the smaller ion replaces the larger ion within the EP macrocycle. As such, negative reaction energies denote a preference for EP complexation with the smaller ion, while positive reaction energies denote selectivity preference for EP complexation of the larger ion.

	La $\rightarrow$ Gd	Gd $\rightarrow$ Lu	La $\rightarrow$ Lu
Pent0	-14.79	-21.18	-6.39
Porph1212	-10.01	-11.79	-1.78
Porph1122	-20.30	-19.47	-0.83
Sapphyrin	-3.31	-4.98	-1.66
Porph2	-5.52	-1.42	4.10
Amethyrin	11.84	16.98	5.14
Isoamethyrin	14.77	24.59	9.82
Pent1	-0.69	-0.65	0.04
Rosarin	4.49	9.11	4.62
Rubyrin	13.02	20.91	7.89
Hex1	6.09	10.44	4.35

Table S6: Calculated energies (*Hartrees*) for each lanthanum complex, including free energy correction utilising the BHLYP/$TZVP_{COSMO}$ model chemistry.

La	TZ(COSMO)	Free energy Corr	Total
Nitrate	-1101.955419	0.070617143	-1101.884802
Pent0	-1379.043032	0.308	-1378.735032
Porph1212	-1606.796001	0.345814684	-1606.450187
Porph1122	-1606.763791	0.346728883	-1606.417062
Sapphyrin	-1533.905271	0.371324636	-1533.533946
Porph2	-1684.111836	0.37693553	-1683.7349
Amethyrin	-1715.837418	0.331013333	-1715.506404
Isoamethyrin	-1715.834256	0.331165714	-1715.503091
Pent1	-1700.010603	0.352049524	-1699.658554
Rosarin	-1627.074323	0.382062661	-1626.692261
Rubyrin	-1793.176166	0.36513094	-1792.811035
Hex1	-1870.531515	0.395619048	-1870.135896

Table S7: Calculated energies (*Hartrees*) for each gadolinium complex, including free energy correction utilising the BHLYP/TZVP$_{\mathrm{COSMO}}$ model chemistry.

Gd	TZ(COSMO)	Free energy Corr	Total
Nitrate	-1759.310262	0.049687619	-1759.260575
Pent0	-2036.41933	0.284952381	-2036.134378
Porph1212	-2264.165976	0.324071993	-2263.841904
Porph1122	-2264.146062	0.320872298	-2263.82519
Sapphyrin	-2191.266184	0.351189411	-2190.914995
Porph2	-2341.473887	0.354423388	-2341.119464
Amethyrin	-2373.170111	0.306807619	-2372.863303
Isoamethyrin	-2373.163375	0.308049524	-2372.855325
Pent1	-2357.366709	0.33128381	-2357.035426
Rosarin	-2284.416701	0.35582516	-2284.060876
Rubyrin	-2450.508484	0.342424531	-2450.16606
Hex1	-2527.874358	0.372392381	-2527.501966

Table S8: Calculated energies (*Hartrees*) for each lutetium complex, including free energy correction utilising the BHLYP/$TZVP_{COSMO}$ model chemistry.

Lu	TZ(COSMO)	Free energy Corr	Total
Nitrate	-2229.46887	0.054510476	-2229.41436
Pent0	-2506.585437	0.287097143	-2506.29834
Porph1212	-2734.324289	0.325759451	-2733.99853
Porph1122	-2734.300675	0.323020665	-2733.977654
Sapphyrin	-2661.42215	0.350717075	-2661.071433
Porph2	-2811.625473	0.358754404	-2811.266718
Amethyrin	-2843.317592	0.308693333	-2843.008899
Isoamethyrin	-2843.302268	0.3088	-2842.993468
Pent1	-2827.52203	0.33288	-2827.18915
Rosarin	-2754.564413	0.357120274	-2754.207292
Rubyrin	-2920.650445	0.343178745	-2920.307267
Hex1	-2998.022323	0.373500952	-2997.648822

Geometries

Macrocycles

Table S9: Optimised geometry of the **pent0** macrocycle with the BHLYP/def2-SVP model chemistry.

Atom	X	Y	Z
C	-2.765649200	-0.956280400	-0.758707500
C	-3.711018600	-0.513886300	0.242116200
C	-3.644864500	0.865964300	0.241871300
C	-2.661637500	1.215569200	-0.759188300
N	-2.265709800	0.108018800	-1.395318100
H	-4.271229900	-1.139599800	0.934407000
H	-4.142686400	1.542601200	0.933998200
C	-1.967692800	-2.173078800	-0.738054000
C	-1.955568100	-3.193974800	0.285983200
N	-0.781861500	-2.152734000	-1.355557100
C	-0.664030600	-3.683147300	0.317869400
H	-2.764169300	-3.443513100	0.970496400
C	0.065214300	-2.943127300	-0.687906900
H	-0.253841100	-4.394246400	1.032562100
C	-1.751127700	2.350600700	-0.739401200
C	-1.640929800	3.365455900	0.284842700
N	-0.573107600	2.217389900	-1.357919200
C	-0.308611000	3.729192900	0.315811700
H	-2.421576600	3.690353600	0.970146600
C	0.345967500	2.923105400	-0.690598200
H	0.168350800	4.397345000	1.030478200

C	1.706469300	2.407705700	-0.657006200
C	2.686553700	2.594672000	0.389753100
N	1.955557300	1.259540900	-1.295522400
C	3.443826500	1.439474800	0.410313700
H	2.741304400	3.422741000	1.093972800
C	2.898473000	0.589428600	-0.624685200
H	4.213514800	1.177161200	1.133783100
C	1.468756900	-2.560182600	-0.654275800
C	2.425478900	-2.838039500	0.393893400
N	1.827082400	-1.442350300	-1.294622500
C	3.289718600	-1.760531800	0.413217700
H	2.399904900	-3.666040200	1.099851700
C	2.828996300	-0.864136500	-0.623803300
H	4.080142200	-1.571451900	1.137197600

Table S10: Optimised geometry of the **porph1212** macrocycle with the BHLYP/def2-SVP model chemistry.

Atom	X	Y	Z
C	3.222673700	1.439225600	0.105367300
C	4.600904600	1.032167900	0.268530000
C	4.569857500	-0.322151400	0.421633200
C	3.181412400	-0.694969200	0.347013000
N	2.402949000	0.410211100	0.154288400
H	5.464913000	1.689278100	0.266065700
H	5.401747400	-1.003406100	0.570275800
C	0.392666700	3.489967200	-0.365320800
C	-0.419167000	4.670000300	-0.565895700
N	-0.342083200	2.399275200	-0.303847400
C	-1.706952600	4.226338000	-0.623581200
H	-0.054277300	5.688870700	-0.649999000
C	-1.639018800	2.798045500	-0.457111700
H	-2.614165900	4.805485300	-0.764396800
C	1.639023600	-2.798052300	0.457048100
C	1.706788400	-4.226114900	0.625547100
N	0.342219000	-2.399460800	0.302218300
C	0.419042000	-4.669830700	0.567401700
H	2.613857700	-4.805066100	0.768087100
C	-0.392599200	-3.490059900	0.364517700
H	0.054055700	-5.688569700	0.652671400
C	-3.181395200	0.694945400	-0.347233200
C	-4.569969500	0.322303200	-0.420309900
N	-2.402774600	-0.410447400	-0.156373700

C	-4.600920400	-1.032145500	-0.268336500
H	-5.402004900	1.003754000	-0.567229900
C	-3.222525400	-1.439425700	-0.107128600
H	-5.464980400	-1.689184900	-0.265242500
C	2.808458800	-2.035079300	0.468799100
H	3.683935100	-2.669501800	0.615489400
C	-2.921623900	-2.852875600	0.079074500
C	-1.838345500	-3.637822800	0.259567500
H	-3.849488700	-3.428598800	0.066655100
H	-2.105796600	-4.692076400	0.357344000
C	1.838527200	3.637575600	-0.261734400
C	2.921850800	2.852568400	-0.081773300
H	2.106010400	4.691784600	-0.359903400
H	3.849776300	3.428209000	-0.070080000
C	-2.808490200	2.035121600	-0.468439000
H	-3.684090000	2.669712400	-0.613657300

Table S11: Optimised geometry of the **porph1122** macrocycle with the BHLYP/def2-SVP model chemistry.

Atom	X	Y	Z
C	3.004822200	1.692515400	0.139902800
C	4.419323500	1.584399300	0.313283900
C	4.673812300	0.246159500	0.483598700
C	3.403369300	-0.404167200	0.408442500
N	2.415765800	0.485020300	0.201557000
H	5.128290600	2.407141800	0.306435900
H	5.631596200	-0.240048200	0.644311700
C	-0.113615000	3.440727200	-0.423274000
C	-1.003188600	4.581281500	-0.677319100
N	-0.780943700	2.322634600	-0.357124100
C	-2.250900200	4.071631900	-0.758564600
H	-0.691648100	5.616016900	-0.775285600
C	-2.109283500	2.639683200	-0.555207900
H	-3.185569900	4.592962200	-0.937344900
C	1.150164300	-3.235049800	0.492188400
C	0.739604300	-4.639357300	0.618823500
N	0.123340200	-2.454722800	0.300977800
C	-0.604606900	-4.648960800	0.491810100
H	1.405820800	-5.480086500	0.782074600
C	-0.994528800	-3.263747700	0.289203200
H	-1.282644000	-5.495346800	0.527026100
C	-3.368924300	0.405870900	-0.409280300
C	-4.652003700	-0.239378100	-0.459380600
N	-2.391199800	-0.479469900	-0.195700800

C	-4.400542500	-1.573264600	-0.262936600
H	-5.609461000	0.246630600	-0.619690700
C	-2.976135100	-1.677289400	-0.103693300
H	-5.111776700	-2.392891400	-0.231831900
C	2.567211500	-2.925982700	0.593218600
C	3.387642000	-1.843165200	0.558468100
H	3.126431300	-3.850540700	0.747695700
H	4.422501700	-2.165151100	0.695768800
C	-2.308765800	-2.914357900	0.116990900
H	-2.990500800	-3.764959800	0.154356900
C	1.309606600	3.702524800	-0.283144400
C	2.463928900	3.018213000	-0.068507900
H	1.489163200	4.774488200	-0.383233900
H	3.303334900	3.717035800	-0.045722500
C	-3.199676900	1.809186100	-0.572019800
H	-4.139814100	2.333815000	-0.746872400

Table S12: Optimised geometry of the **sapphyrin** macrocycle with the BHLYP/def2-SVP model chemistry.

Atom	X	Y	Z
C	0.180796600	-2.444305100	2.792541100
C	0.235803300	-3.876446200	2.979581000
C	0.211850500	-4.421562500	1.718416700
C	0.143148100	-3.299825100	0.808436500
N	0.126875300	-2.159052800	1.474411100
H	0.285505300	-4.402351400	3.931212500
H	0.238532500	-5.474707000	1.451600400
C	0.182640200	-1.491708100	3.825492100
H	0.231286800	-1.914060800	4.832212000
C	0.132704400	-0.091838800	3.794298800
C	0.146118500	0.711649200	5.003428500
N	0.067097700	0.713633100	2.707059100
C	0.086557200	2.008989900	4.593011800
H	0.194497400	0.330909700	6.021459000
C	0.037687400	1.971020800	3.133986700
H	0.076303600	2.906227300	5.208208300
C	-0.030700700	3.170847300	2.368132600
H	-0.040762900	4.068696200	2.989791300
C	-0.087288500	3.450416600	0.999899300
C	-0.151219700	4.851453700	0.551542500
N	-0.095217300	2.631834700	-0.066504700
C	-0.195828700	4.815899100	-0.795668500
H	-0.159182800	5.722306500	1.203056100
C	-0.159161600	3.393113200	-1.172841400

H	-0.248413700	5.651171100	-1.490239600
C	-0.192431100	3.041697500	-2.525011300
H	-0.245012300	3.905532300	-3.191097200
C	-0.172652500	1.803044900	-3.229442400
C	-0.219964800	1.763856900	-4.688322700
N	-0.113122600	0.569928300	-2.739541700
C	-0.185263300	0.446456200	-5.032718100
H	-0.272290700	2.627285600	-5.348087100
C	-0.117685500	-0.291892100	-3.784673600
H	-0.203312600	0.012475200	-6.030222700
C	0.095214200	-3.337934600	-0.640803300
C	0.105353000	-4.506289200	-1.493029800
N	0.033333300	-2.233874100	-1.363369400
C	0.045289600	-4.028437000	-2.779895100
H	0.151097800	-5.543907900	-1.173250500
C	0.000528400	-2.588238000	-2.665385100
H	0.032953900	-4.603670700	-3.703802300
C	-0.067366400	-1.691646700	-3.744938400
H	-0.084297300	-2.166697000	-4.728932400

Table S13: Optimised geometry of the **porph2** macrocycle with the BHLYP/def2-SVP model chemistry.

Atom	X	Y	Z
C	3.143692200	1.647822500	0.626570300
C	4.241461500	1.609722800	1.581940900
C	4.352541900	0.318881100	1.965529900
C	3.340285600	-0.408902800	1.212756200
N	2.617144700	0.427287200	0.454419700
H	4.832101100	2.463750200	1.896182500
H	5.060207100	-0.123021500	2.659056600
C	0.614414500	3.308484400	-1.215463400
C	0.038560300	4.289225300	-2.124650100
N	-0.347931500	2.597746400	-0.609225900
C	-1.301332100	4.174056100	-1.992159400
H	0.600996700	4.981464600	-2.742239300
C	-1.515224300	3.104277900	-1.028389200
H	-2.082739100	4.745225600	-2.482179200
C	1.606625800	-3.227297800	-0.055494300
C	1.531516700	-4.489170400	-0.776470700
N	0.395277400	-2.661725200	0.046259400
C	0.228253200	-4.656581100	-1.093226900
H	2.371265500	-5.140739700	-0.994143500
C	-0.468638900	-3.508120100	-0.531884600
H	-0.239615600	-5.480232500	-1.622224900
C	-3.485623300	0.607640700	0.533965100
C	-4.619968500	0.046486400	1.253045700
N	-2.664598100	-0.362691500	0.106409200

C	-4.472237300	-1.295536900	1.187238700
H	-5.422856700	0.617722400	1.707030700
C	-3.235001600	-1.523864000	0.456031500
H	-5.122337700	-2.069333100	1.581904600
C	2.833829200	-2.892524900	0.566772900
C	3.428418900	-1.821692000	1.202600900
H	3.481035400	-3.772171600	0.597234900
H	4.316978700	-2.137591500	1.752585700
C	-2.928657900	-2.852961000	0.071658800
C	-1.881942000	-3.573654400	-0.464769800
H	-3.814657000	-3.481350400	0.189443100
H	-2.220578700	-4.548044700	-0.822330300
C	1.997516400	3.413894000	-0.936024100
C	2.928596200	2.852049100	-0.086078400
H	2.408763600	4.288547600	-1.443581600
H	3.792730600	3.509347500	0.034985500
C	-3.543262800	1.981698900	0.197761600
C	-2.833330800	2.895726300	-0.552978800
H	-4.502683700	2.396028400	0.513440200
H	-3.458995600	3.750121700	-0.821310400

Table S14: Optimised geometry of the **amethyrin** macrocycle with the BHLYP/def2-SVP model chemistry.

Atom	X	Y	Z
C	2.955424000	-2.867068600	0.066041900
C	3.016460500	-4.329538900	0.075939100
C	1.728570300	-4.756687000	0.043800400
C	0.911980700	-3.543708200	0.014583800
N	1.679620500	-2.464673900	0.028274900
H	3.917727400	-4.933696000	0.104343400
H	1.367413500	-5.778867900	0.040540800
C	4.071395200	-0.610120000	0.095959500
C	5.270295000	0.228611500	0.138360800
N	2.977606700	0.160183400	0.061023800
C	4.828168200	1.511788300	0.128153800
H	6.297111400	-0.121503500	0.172118300
C	3.369040100	1.425173600	0.079057900
H	5.421080500	2.419077400	0.151788200
C	2.433896800	2.498108100	0.055310100
C	2.703555400	3.930706200	0.074791200
N	1.124378800	2.272979500	0.013593900
C	1.483015500	4.534869600	0.042998900
H	3.676941800	4.407223500	0.108399300
C	0.508589100	3.451081100	0.005373800
H	1.270875300	5.598225700	0.045786500
C	-0.911471300	3.543805500	-0.031345900
C	-1.728456400	4.756777300	-0.042863500
N	-1.678848800	2.464764600	-0.056864600

C	-3.016395900	4.329704700	-0.076138200
H	-1.367592100	5.778949600	-0.027378800
H	-3.917940800	4.933948700	-0.093106100
C	-0.508083500	-3.451149900	-0.020284200
C	-1.483090300	-4.534967800	-0.032402100
N	-1.123636200	-2.273012500	-0.042559000
C	-2.703763800	-3.930897500	-0.061976500
H	-1.271284000	-5.598320900	-0.019369500
H	-3.677599300	-4.407512100	-0.077846100
C	-2.433577900	-2.498242600	-0.067162600
C	-2.955029200	2.867257600	-0.083375100
C	-5.270723400	-0.228612800	-0.128060800
C	-4.828619900	-1.511846300	-0.115644200
C	-4.071262100	0.610136200	-0.109598200
H	-6.297981900	0.121490200	-0.146521100
C	-3.368884500	-1.425289800	-0.090604700
N	-2.976973200	-0.160245300	-0.088788000
H	-5.422020000	-2.419148900	-0.122089700
C	4.065520700	-2.011800700	0.096482900
C	-4.065288600	2.011896700	-0.109037000
H	5.041541700	-2.494807800	0.127568800
H	-5.041686000	2.494960000	-0.127276100

Table S15: Optimised geometry of the **isoamethyrin** macrocycle with the BHLYP/def2-SVP model chemistry.

Atom	X	Y	Z
C	3.820871000	-0.325336400	-0.055466200
C	5.082839300	-1.031964100	-0.078692300
C	4.756796100	-2.360806900	-0.104378900
C	3.307359400	-2.413601900	-0.094918900
N	2.803412400	-1.175428400	-0.066224800
H	6.075095400	-0.593625900	-0.076587200
H	5.433812300	-3.209202700	-0.126713100
C	3.546796500	1.085105300	-0.022209200
C	4.467515700	2.234710500	0.007958800
N	2.307383300	1.498161900	-0.009854000
C	3.680912200	3.340591100	0.040430500
H	5.551333500	2.197565700	0.005924400
C	2.303578500	2.843326300	0.029416900
H	3.997470500	4.377127600	0.069932500
C	1.071810100	3.498576300	0.057955800
C	0.715326300	4.917981700	0.106789300
N	-0.046762800	2.750236900	0.045871800
C	-0.641717600	4.952308200	0.123023000
H	1.398637400	5.759559200	0.126366800
C	-1.081641900	3.546908400	0.084267000
H	-1.277612500	5.830117200	0.158569100
C	-2.405000500	2.985912600	0.088286600
C	-3.696194600	3.637320400	0.122287100
N	-2.541886400	1.667442600	0.059018100

C	-4.616483900	2.624511200	0.111030300
H	-3.886802000	4.704883400	0.150230000
H	-5.698504400	2.711683900	0.128437800
C	1.143363200	-3.760099100	-0.099150500
C	0.510472200	-5.068761000	-0.119618000
N	0.188518500	-2.788972300	-0.067354100
C	-0.827356300	-4.839259800	-0.099319300
H	1.024731300	-6.024099400	-0.145909600
H	-1.624382200	-5.573084900	-0.105702000
C	-0.979735400	-3.387415300	-0.066475600
C	-3.850696500	1.393365500	0.071328500
C	-4.490563700	-2.408948200	-0.002991100
C	-3.552606300	-3.389845300	-0.035689900
C	-3.758594300	-1.153073600	0.015293700
H	-5.570449500	-2.516541500	0.007735300
C	-2.263090100	-2.704932100	-0.036401600
N	-2.419452500	-1.401959600	-0.006260800
H	-3.716149400	-4.460635200	-0.056893400
C	-4.384352200	0.088486200	0.049981000
H	-5.473794000	0.041571900	0.063224400
C	2.522944600	-3.585229300	-0.110795500
H	3.092848900	-4.514630700	-0.135752500

Table S16: Optimised geometry of the **pent1** macrocycle with the BHLYP/def2-SVP model chemistry.

Atom	X	Y	Z
C	0.017022500	-2.352380300	3.029225600
C	0.004487800	-3.727870600	3.529873100
C	-0.023169700	-4.518174100	2.437795500
C	-0.026808900	-3.612649800	1.287608100
N	-0.002534200	-2.335963000	1.690248700
H	0.015510600	-4.018486100	4.575075700
H	-0.039545700	-5.601788600	2.387091800
C	0.046230400	-1.289030700	3.940143200
H	0.058819700	-1.627710800	4.975529700
C	0.062799300	0.107469100	3.833247200
C	0.094922100	0.921244000	5.049540700
N	0.052942500	0.885477800	2.743417500
C	0.103590700	2.204352500	4.635304400
H	0.108366500	0.537844900	6.064363500
C	0.076430600	2.153601900	3.172627000
H	0.125792400	3.108579800	5.234394200
C	0.076926500	3.349138200	2.442655200
H	0.097947500	4.229084300	3.084636700
C	0.056224600	3.679208100	1.081867400
C	0.061987800	5.087840300	0.683569600
N	0.030562800	2.883136700	0.005180100
C	0.038476400	5.090607700	-0.664495500
H	0.081212200	5.934613000	1.361537800
C	0.019382200	3.683526800	-1.068063300

H	0.034197800	5.940091200	-1.339355400
C	-0.005165300	3.358923900	-2.430595500
C	-0.051962300	-4.145728900	-0.007653700
H	-0.067191200	-5.235093900	-0.009650800
H	-0.007913200	4.241531300	-3.069290300
C	-0.025677000	2.166413300	-3.164585900
C	-0.045346700	2.223003700	-4.627463200
N	-0.030219200	0.896286400	-2.740147900
C	-0.060808100	0.941693300	-5.046468700
H	-0.046391400	3.129785300	-5.223127600
C	-0.051350100	0.122719400	-3.832876100
H	-0.077397000	0.562251400	-6.062756300
C	-0.061192600	-3.607507300	-1.300466500
C	-0.085897400	-4.508591100	-2.454094700
N	-0.050275000	-2.328969500	-1.698899300
C	-0.088547700	-3.714109000	-3.543326000
H	-0.098610100	-5.592416600	-2.407033600
C	-0.066213800	-2.340276000	-3.037682700
H	-0.104205200	-4.000324900	-4.589706900
C	-0.065679700	-1.273333900	-3.945266800
H	-0.081731400	-1.608018900	-4.981926100

Table S17: Optimised geometry of the **rosarin** macrocycle with the BHLYP/def2-SVP model chemistry.

Atom	X	Y	Z
C	2.253886600	3.147915700	-0.083448400
C	2.535972400	4.566161700	-0.131967500
C	1.314443200	5.186378100	-0.176107800
C	0.333690900	4.132119700	-0.153428200
N	0.960148200	2.921732800	-0.097203900
H	3.516119300	5.032317200	-0.132774600
H	1.107628500	6.252979900	-0.219443000
C	-1.045240800	4.361657400	-0.185739300
H	-1.299722100	5.423481100	-0.231159600
C	-2.170426200	3.531148000	-0.173027300
C	-3.522174900	4.025498600	-0.216615500
N	-2.179984600	2.168336400	-0.119633400
C	-4.329756500	2.918156700	-0.187882000
H	-3.821549400	5.069772100	-0.263018300
C	-3.435600000	1.782552200	-0.127026800
H	-5.414572800	2.888829000	-0.206554000
C	-3.853402900	0.375046800	-0.078468200
C	-5.223243500	-0.090628900	-0.082544600
N	-3.011282300	-0.631485100	-0.024751800
C	-5.150653200	-1.458252100	-0.026814600
H	-6.116656400	0.524410200	-0.121659800
C	-3.747280300	-1.779450600	0.008669600
H	-5.971715600	-2.170952200	-0.011694800
C	3.264475400	2.083362600	-0.025675500

C	4.696153700	2.290045000	-0.008549200
N	2.970142900	0.803918900	0.017971800
C	5.251416700	1.037999800	0.049067400
H	5.213853200	3.243580700	-0.035938300
C	4.146317100	0.114956900	0.064925800
H	6.306238300	0.775652400	0.077763000
C	4.302692200	-1.273730800	0.120684900
C	-3.257529400	-3.087861100	0.067415400
H	5.350204600	-1.583841800	0.150152100
H	-4.050574200	-3.839572000	0.083956700
C	3.413511700	-2.352568100	0.146882300
C	3.836138100	-3.728025400	0.205070600
N	2.051110300	-2.290224200	0.122488300
C	2.687278700	-4.475510000	0.214288200
H	4.863906400	-4.081747400	0.234738100
C	1.599538500	-3.522963100	0.161198800
H	2.600556200	-5.556686900	0.253230600
C	-1.975910200	-3.646092800	0.109274700
C	-1.729211100	-5.063600800	0.166266300
N	-0.790155400	-2.972213100	0.102843500
C	-0.366280600	-5.208364100	0.192986000
H	-2.484601500	-5.845519800	0.183539300
C	0.171224600	-3.865998600	0.151293200
H	0.200876200	-6.132721200	0.236420300

Table S18: Optimised geometry of the **rubyrin** macrocycle with the BHLYP/def2-SVP model chemistry.

Atom	X	Y	Z
C	2.253054800	3.846198200	-0.054459900
C	2.273040400	5.286538300	-0.089810400
C	0.964920100	5.672473000	-0.116976200
C	0.198756000	4.452326700	-0.097583800
N	0.998257400	3.378525400	-0.060199500
H	3.159740400	5.911526600	-0.093292400
H	0.560065500	6.678482800	-0.147191200
C	-1.221822800	4.493272800	-0.118112800
H	-1.590736700	5.518809200	-0.146549800
C	-2.244733500	3.575574100	-0.111498300
C	-3.632451500	4.000252900	-0.140000100
N	-2.179087500	2.202649600	-0.079802700
C	-4.371842200	2.867289200	-0.124392800
H	-3.977560300	5.028038200	-0.167955200
C	-3.409639200	1.769596600	-0.086680900
H	-5.450565500	2.781475500	-0.137311800
C	3.468169900	3.109485700	-0.018121700
H	4.335223100	3.770424600	-0.020014800
C	-3.824601000	0.365007900	-0.060715200
C	-5.229237600	-0.034297200	-0.067975700
N	-3.026763600	-0.666581300	-0.029374600
C	-5.224221200	-1.386866300	-0.038766800
H	-6.088180400	0.623574600	-0.091752900
C	-3.828134300	-1.783756700	-0.014428200

H	-6.072478000	-2.062611000	-0.033529600
C	3.828062800	1.783853100	0.018926200
C	5.224013000	1.387157500	0.052705800
N	3.026742400	0.666596400	0.029960000
C	5.228994500	0.034635000	0.083921400
H	6.072192200	2.063018600	0.052428100
C	3.824472500	-0.364857800	0.067749900
H	6.087827700	-0.623108300	0.114545900
C	-3.468138700	-3.109495400	0.017764700
H	-4.335219200	-3.770403700	0.020682000
C	3.409549000	-1.769513300	0.091079000
C	4.371752100	-2.867171700	0.129999600
N	2.179070800	-2.202681600	0.079744600
C	3.632443100	-4.000242400	0.140871200
H	5.450427600	-2.781242800	0.146271400
C	2.244772000	-3.575651100	0.109427200
H	3.977543600	-5.028065000	0.167500000
C	-2.252940600	-3.846315100	0.048355300
C	-2.272817700	-5.286834100	0.075599800
N	-0.998176700	-3.378577600	0.057430200
C	-0.964688000	-5.672804900	0.101441000
H	-3.159519800	-5.911832700	0.075002900
C	-0.198620600	-4.452504000	0.089296100
H	-0.559706300	-6.678906500	0.126308600
C	1.221929900	-4.493436500	0.111730400
H	1.590861900	-5.519025500	0.137755500

Table S19: Optimised geometry of the **hex1** macrocycle with the BHLYP/def2-SVP model chemistry.

Atom	X	Y	Z
C	4.107530200	1.688351700	-1.018853800
C	4.915292400	2.788593100	-1.551439700
C	4.126657900	3.875158100	-1.516496300
C	2.847099300	3.425472900	-0.962861300
N	2.876349200	2.109975300	-0.698345100
H	5.942784200	2.705754400	-1.886705000
H	4.356630300	4.891140500	-1.816329500
C	1.880309600	4.409180200	-0.723400900
H	2.278938300	5.394450900	-0.958354800
C	0.575869900	4.521632900	-0.220840500
C	0.092303400	5.873034200	0.067707900
N	-0.363932400	3.607000500	0.056497400
C	-1.150178300	5.722479000	0.557216100
H	0.659095400	6.785682400	-0.076637400
C	-1.428706100	4.285826200	0.508931100
H	-1.842972200	6.484285100	0.895842300
C	-2.729149300	3.884785100	0.843590700
C	4.750648800	0.453746600	-0.852947300
H	-3.309617900	4.739060200	1.186572600
H	5.803147500	0.539125100	-1.117071900
C	-3.540981900	2.745503200	0.770595100
C	-4.969587200	2.937554600	1.028148900
N	-3.284670100	1.475929000	0.419984000
C	-5.553036400	1.755963900	0.764841800

H	-5.431277300	3.868523100	1.336586800
C	-4.465916300	0.843586100	0.404725900
H	-6.602460300	1.489943700	0.819300000
C	4.467704000	-0.841521300	-0.401342500
C	5.610457100	-1.735130300	-0.197550900
N	3.315773200	-1.463189300	-0.107845200
C	5.097840200	-2.889971600	0.258489800
H	6.648294700	-1.474098100	-0.370100400
C	3.644388200	-2.705003400	0.275272800
H	5.616838800	-3.800398500	0.535580200
C	2.871244100	-3.829815500	0.594376600
C	-4.821600400	-0.481140400	0.110755800
H	3.514468600	-4.660479600	0.878252400
H	-5.904550000	-0.576597900	0.056129200
C	1.533192200	-4.243559100	0.556653200
C	1.280078300	-5.670482900	0.774069300
N	0.394997900	-3.593921700	0.272732000
C	-0.033304900	-5.848588900	0.556275700
H	2.032271100	-6.408708400	1.027554600
C	-0.575236700	-4.519593100	0.261907700
H	-0.610993100	-6.764710500	0.601272600
C	-4.214586100	-1.728548100	-0.074927000
C	-5.100854600	-2.861579300	-0.353652800
N	-2.940832000	-2.135267600	0.041609400
C	-4.313035000	-3.948840500	-0.376158800
H	-6.173520000	-2.797030900	-0.495836600
C	-2.954483200	-3.466524600	-0.112976600

H	-4.588795200	-4.984040000	-0.541603100
C	-1.952543000	-4.437817700	0.018975400
H	-2.383385200	-5.435179100	-0.048170100

La-Complexes

Table S20: Optimised geometry of the **La$^{(III)}$-pent0** complex with the BHLYP/def2-SVP model chemistry.

Atom	X	Y	Z
C	2.7895121	0.9768285	-0.3741031
C	3.7741183	0.4914150	0.5351746
C	3.7055933	-0.8857922	0.5043897
C	2.6789169	-1.2296923	-0.4235426
N	2.2432047	-0.0896085	-1.0243041
H	4.3847957	1.0915131	1.1960819
H	4.2521900	-1.5725320	1.1363636
C	2.0007662	2.1697578	-0.3460290
C	1.9929364	3.2698698	0.5707310
N	0.7847969	2.0953006	-0.9356985
C	0.7083836	3.7603788	0.5914528
H	2.8187168	3.5935320	1.1896677
C	-0.0567637	2.9557105	-0.3121840
H	0.3264372	4.5451012	1.2303166
C	1.7806427	-2.3447753	-0.4456965
C	1.6534516	-3.4486466	0.4486085
N	0.5795051	-2.1395997	-1.0500607
C	0.3226622	-3.8079979	0.4544010
H	2.4350739	-3.8583680	1.0738692
C	-0.3503689	-2.9201327	-0.4361546
H	-0.1412555	-4.5542163	1.0850762
C	-1.6874388	-2.4079646	-0.4029816

C	-2.7473050	-2.6376043	0.5263289
N	-1.8854748	-1.1996991	-0.9892912
C	-3.5007043	-1.4846642	0.5672000
H	-2.8746780	-3.5139850	1.1471805
C	-2.8948893	-0.5610379	-0.3368767
H	-4.3338379	-1.2813789	1.2262645
C	-1.4363489	2.5824611	-0.2994781
C	-2.4887194	2.8933756	0.6210276
N	-1.7400484	1.4149706	-0.9153815
C	-3.3511268	1.8222493	0.6157848
H	-2.5445428	3.7647687	1.2592689
C	-2.8179529	0.8664969	-0.3082581
H	-4.2178624	1.6867809	1.2483769
La	-0.0366244	0.0698343	-2.1595110
O	-0.9005676	1.5048640	-4.2689791
H	-1.2569718	2.3843354	-4.1486044
H	-1.6486365	0.9203631	-4.4444108
O	0.3610807	-2.1213458	-3.9006176
H	0.6058909	-2.5792188	-3.0832553
H	1.1846940	-1.8483954	-4.3103915
O	2.2195332	0.0622496	-3.7941558
H	2.6692817	0.0357227	-2.9311921
H	2.6151800	0.7736441	-4.2951361
O	-2.1454897	-0.8676980	-3.8769628
H	-1.6032107	-1.5938647	-4.2000623
H	-2.5354254	-1.1840282	-3.0522469

Table S21: Optimised geometry of the **La$^{(III)}$-porph1212** complex with the BHLYP/def2-SVP model chemistry.

Atom	X	Y	Z
C	3.095065800	1.481647300	-0.041843200
C	4.344952200	1.194812300	-0.664071000
C	4.271073600	-0.105332000	-1.073146400
C	2.998812000	-0.592597600	-0.656028000
N	2.293335700	0.423715900	-0.042919100
H	5.164291900	1.890709400	-0.783779900
H	5.025115900	-0.687924800	-1.586414200
C	0.467449000	3.386120800	-0.196832100
C	-0.149877300	4.427231000	-0.949743300
N	-0.262549300	2.276596600	-0.207794300
C	-1.295696500	3.889835400	-1.465038600
H	0.247446300	5.422903500	-1.093964400
C	-1.371524400	2.551272200	-0.983243700
H	-2.025856900	4.365943200	-2.106931000
C	1.565216500	-2.670958800	-0.272968700
C	1.521751800	-4.085870500	-0.357982300
N	0.443294200	-2.206514800	0.376572300
C	0.373222200	-4.481680200	0.275770300
H	2.274076100	-4.708316900	-0.824836200
C	-0.276794200	-3.289234900	0.686002200
H	-0.002590400	-5.486356400	0.415175500
C	-2.842374900	0.465946300	-0.771391300
C	-4.108511600	-0.101142000	-1.087643300
N	-2.154551800	-0.377138500	0.080053000

C	-4.191947000	-1.280005700	-0.401183000
H	-4.846667000	0.343451000	-1.742780800
C	-2.955213900	-1.421421200	0.290237700
H	-5.005542500	-1.992765500	-0.389282400
C	2.649014000	-1.928660600	-0.743084600
H	3.396910500	-2.537473000	-1.243459000
C	-2.694914900	-2.586245900	1.129646000
C	-1.593446200	-3.339537300	1.303876800
H	-3.598699500	-2.978342800	1.595016000
H	-1.739954500	-4.236118300	1.903340300
C	1.741771400	3.579280400	0.488392800
C	2.823945100	2.790131800	0.547484000
H	1.860479500	4.576414400	0.910051100
H	3.701167700	3.229269900	1.019597200
C	-2.462846200	1.726939400	-1.224461000
H	-3.203338400	2.182117400	-1.876782700
O	-1.876136900	-0.575550400	3.290730500
H	-1.187799500	-0.798416600	3.939897600
H	-2.271566200	-1.407779900	3.001517400
O	-1.972754800	1.834847900	2.199103900
H	-2.399967900	1.133737700	2.717173200
H	-2.543276800	2.062284600	1.458258300
N	1.793937700	-0.620528800	3.859026800
O	0.621311500	-1.041755000	3.644002000
O	2.294140500	0.044442900	2.934815900
O	2.358534500	-0.845683700	4.879216800
O	0.433550900	2.064543700	3.162141100

H	1.273359600	2.496035400	3.342241500
H	-0.283087500	2.706432700	3.093061100
La	0.107339300	0.189511200	1.370934500

Table S22: Optimised geometry of the **La$^{(III)}$-porph1122** complex with the BHLYP/def2-SVP model chemistry.

Atom	X	Y	Z
C	3.170705000	1.616858500	-0.240227400
C	4.573407300	1.455636600	-0.228146700
C	4.815863300	0.101633000	-0.095513200
C	3.555331700	-0.531158900	-0.030167200
N	2.581455400	0.404519800	-0.118974600
H	5.303094200	2.251405000	-0.303011500
H	5.776671700	-0.393966500	-0.045235800
C	-0.056531800	3.418546300	-0.344001700
C	-0.906623900	4.593197600	-0.258199300
N	-0.792567400	2.327067700	-0.289669900
C	-2.176955300	4.150258800	-0.125049700
H	-0.556276500	5.616304000	-0.279584200
C	-2.116237700	2.708294600	-0.121297200
H	-3.080032800	4.736221900	-0.014503600
C	1.152731900	-3.320302100	0.316547100
C	0.754273900	-4.671588400	0.663616700
N	0.085054200	-2.559592800	0.172038500
C	-0.596089200	-4.673686200	0.746494500
H	1.434060900	-5.494693800	0.837848800
C	-1.032966800	-3.329381300	0.460046800
H	-1.247350300	-5.499079400	1.002859100
C	-3.371270700	0.485957200	0.232139600
C	-4.634033500	-0.132532400	0.422781900
N	-2.391708100	-0.448621700	0.242188400

C	-4.390850000	-1.480847600	0.553065200
H	-5.588068700	0.376417500	0.465923400
C	-2.986778100	-1.648581400	0.437144400
H	-5.110894700	-2.271142300	0.720337800
C	2.578492900	-3.021811500	0.168980400
C	3.457775300	-1.975993500	0.071318700
H	3.118102700	-3.963366400	0.224098200
H	4.474410700	-2.362884500	0.055763200
C	-2.337854900	-2.918622900	0.546937600
H	-3.030786200	-3.724084200	0.774353300
C	1.389149800	3.607486500	-0.477251300
C	2.577802100	2.932069100	-0.406011600
H	1.568348300	4.669890100	-0.618050300
H	3.398722000	3.636025200	-0.525030000
C	-3.203176200	1.899846400	0.084243300
H	-4.138641700	2.444928900	0.175658900
O	0.865900800	1.210524600	-2.530568400
H	1.291983700	2.066301400	-2.400209800
H	1.543623100	0.550840800	-2.735576900
O	1.364183300	-1.373016000	-2.258114000
H	0.620846000	-1.874552500	-2.611494700
H	2.028633300	-1.999461400	-1.941437600
N	0.726671800	0.352424400	2.831627900
O	0.741745700	-0.777952200	2.285125600
O	0.372471600	1.298698400	2.086361200
O	1.022196200	0.513954100	3.969806000
La	0.143232300	-0.031702500	-0.125733900

O	-1.211648500	-0.412347200	-2.449873800
H	-0.890002200	0.379655200	-2.906019700
H	-2.160952800	-0.340412800	-2.310608500

Table S23: Optimised geometry of the **La$^{(III)}$-sapphyrin** complex with the BHLYP/def2-SVP model chemistry.

Atom	X	Y	Z
C	0.209092500	-2.305185300	2.946877900
C	0.232588500	-3.716812800	3.175505400
C	0.220386100	-4.320179700	1.946867200
C	0.199524000	-3.264662600	0.991072900
N	0.182866400	-2.063193400	1.607573500
H	0.257553200	-4.193595200	4.147042700
H	0.236343800	-5.379109100	1.727170400
C	0.239452900	-1.335041800	3.949139300
H	0.313715000	-1.723003700	4.960439500
C	0.189658400	0.051271200	3.865190400
C	0.259728800	0.880085800	5.032151800
N	0.031273000	0.837926000	2.745681900
C	0.136625700	2.165061300	4.613430500
H	0.383694400	0.515159800	6.043598700
C	-0.008911400	2.124179200	3.185712100
H	0.139504900	3.067728500	5.210215100
C	-0.214300700	3.303962600	2.446450000
H	-0.260292600	4.193198800	3.066598900
C	-0.343257700	3.566614100	1.090686000
C	-0.501300700	4.921778300	0.604251700
N	-0.298971100	2.705125900	0.032816400
C	-0.516209000	4.857263700	-0.742712200
H	-0.572128000	5.798474800	1.234943200
C	-0.367802900	3.461335400	-1.099793400

H	-0.604282300	5.669295800	-1.452789100
C	-0.264736400	3.067386700	-2.424879100
H	-0.349224400	3.889288700	-3.128312100
C	-0.060213800	1.823210500	-3.051119000
C	0.006065100	1.744570900	-4.488615200
N	0.085760600	0.595686800	-2.503336600
C	0.183680000	0.439607500	-4.797701500
H	-0.083761000	2.588504300	-5.159950600
C	0.222839000	-0.281720900	-3.553987600
H	0.270540700	-0.012831900	-5.777101300
C	0.239041300	-3.340428200	-0.427599000
C	0.225525400	-4.474801600	-1.284184800
N	0.314173300	-2.202167300	-1.156126900
C	0.286427200	-3.985971200	-2.565866000
H	0.171572400	-5.509330900	-0.973141500
C	0.327101900	-2.562658700	-2.467142700
H	0.289436300	-4.552208300	-3.488454000
C	0.330905300	-1.665419500	-3.539709800
H	0.387181900	-2.123626000	-4.522316500
O	-2.347595600	0.847487200	1.302220100
H	-2.454707200	1.775802500	1.065286900
H	-1.860754600	0.843252600	2.145503900
O	2.578705000	-1.105410300	0.091176100
H	2.187633100	-1.764874800	-0.506662100
H	2.680831600	-1.548642300	0.940265600
La	0.071590700	0.104577200	0.118255500
O	2.264084500	1.472065100	-0.159760300

H	3.021970100	0.940323200	-0.424885300
H	2.256411600	2.320542500	-0.611103800
O	-2.215640700	-0.896125800	-0.616810400
H	-2.277938700	-1.793051900	-0.955440300
H	-2.896868100	-0.719047100	0.041656100

Table S24: Optimised geometry of the $\mathbf{La}^{(III)}$**-porph2** complex with the BHLYP/def2-SVP model chemistry.

Atom	X	Y	Z
C	2.959633100	1.477989000	-0.430734400
C	4.327618200	1.618628200	0.038040700
C	4.481290700	0.758469900	1.060602500
C	3.250397900	-0.004489800	1.126693400
N	2.321712600	0.509831200	0.283377800
H	5.051331200	2.307403400	-0.377565300
H	5.361635300	0.567588100	1.659451500
C	0.017134600	2.763809900	-1.632036000
C	-0.810168200	3.793862200	-2.200603500
N	-0.606908800	2.226881700	-0.528956800
C	-1.867015000	3.949272900	-1.365083900
H	-0.567901900	4.364814000	-3.087543200
C	-1.734681000	2.927653400	-0.367522200
H	-2.688793300	4.648988900	-1.437260800
C	1.641310800	-2.863429600	0.658609400
C	1.705913700	-4.206301400	0.168567000
N	0.615215900	-2.183528800	0.043244000
C	0.786784200	-4.294025700	-0.824654300
H	2.412817700	-4.955524700	0.499846300
C	0.081266500	-3.042816800	-0.843944100
H	0.565346700	-5.140766500	-1.460397200
C	-3.472414100	0.340284300	0.402925400
C	-4.676310200	-0.370330000	0.192859100
N	-2.426705500	-0.291188100	-0.184539700

C	-4.354079800	-1.416085800	-0.639864500
H	-5.654693200	-0.077545900	0.550675200
C	-2.949883300	-1.364761400	-0.834112400
H	-5.019996500	-2.152106900	-1.071945000
C	2.619077800	-2.427089800	1.553374900
C	3.254433400	-1.219747200	1.814211000
H	3.090417100	-3.260910900	2.067533700
H	4.071127200	-1.301855800	2.524421700
C	-2.300263600	-2.358548900	-1.626351300
C	-1.086545000	-2.976010300	-1.668900900
H	-3.007976600	-2.803494700	-2.323459300
H	-1.055055600	-3.765160400	-2.415366100
C	1.336397900	2.680199400	-2.057295900
C	2.545804500	2.256930200	-1.506678700
H	1.488774800	3.259467100	-2.964355300
H	3.395897100	2.709126400	-2.010937700
C	-3.474646500	1.654912800	0.969616300
C	-2.759146900	2.752873900	0.630865700
H	-4.304067900	1.833611500	1.648935700
H	-3.131700500	3.692816600	1.033750800
N	1.040186500	-0.708885400	3.945383500
O	0.391337700	-1.519292400	3.256384300
O	1.081275300	0.461720100	3.497291800
O	-1.805478900	0.448627800	3.155297400
H	-2.115467400	-0.464563300	3.255692700
H	-1.401552500	0.747235200	3.974979100
O	1.580704400	-1.017352600	4.961089400

La	-0.186092400	0.107162700	1.115322700
O	-1.869959300	-1.969017900	2.046115000
H	-1.285203700	-2.672160300	2.348597100
H	-2.456044900	-2.308883300	1.359399300
O	0.409502400	2.484667100	2.096371600
H	1.086965700	2.417686300	2.779202000
H	0.639660000	3.166043500	1.458998500

Table S25: Optimised geometry of the **La$^{(III)}$-amethyrin** complex with the BHLYP/def2-SVP model chemistry.

Atom	X	Y	Z
C	2.785253200	-2.806056100	0.127950000
C	2.893641400	-4.253627700	0.155254300
C	1.630987300	-4.731268900	0.199806000
C	0.779340100	-3.565202500	0.188637500
N	1.488473100	-2.427247600	0.141265700
H	3.821088600	-4.809697700	0.148272200
H	1.302422900	-5.759972100	0.233456600
C	3.915716600	-0.584563800	0.157409300
C	5.124771300	0.209071200	0.102189000
N	2.831082400	0.219712800	0.260587300
C	4.739119400	1.505869100	0.164434100
H	6.127741500	-0.186088500	0.016045200
C	3.301691300	1.478306700	0.256152900
H	5.358991100	2.390472200	0.136779100
C	2.409271900	2.560013400	0.298435600
C	2.663718600	3.976303800	0.303751500
N	1.090979100	2.320458600	0.291488900
C	1.441993200	4.577341600	0.287453500
H	3.634266600	4.451405600	0.310548100
C	0.477706400	3.509768600	0.265386400
H	1.225906800	5.635998300	0.276320500
C	-0.920964800	3.548949100	0.170769100
C	-1.773832400	4.708081900	0.128991400
N	-1.627632100	2.407882700	0.090595200

C	-3.033901800	4.227542100	0.021036600
H	-1.449710800	5.737637200	0.175745900
H	-3.959610400	4.783313800	-0.037937400
C	-0.620826900	-3.528642600	0.204954400
C	-1.581634100	-4.603771300	0.204583100
N	-1.245501900	-2.346821200	0.178052100
C	-2.806154700	-4.013463200	0.157805800
H	-1.357737800	-5.660618400	0.225706800
H	-3.772970800	-4.495395900	0.134262100
C	-2.559059000	-2.594306300	0.132966400
C	-2.926400500	2.784332600	0.000155100
C	-5.252963600	-0.228007300	-0.089047700
C	-4.878096500	-1.526790500	-0.016551400
C	-4.036916900	0.555663700	-0.066326100
H	-6.253812200	0.177360300	-0.146225200
C	-3.441045900	-1.510130600	0.043957400
N	-2.956229800	-0.254461300	0.005628100
H	-5.505033200	-2.406635500	-0.003923900
La	-0.187791200	0.023773000	-0.189083000
N	0.042712600	0.002719200	-3.170699700
O	0.424531100	0.971005400	-2.460957100
O	0.126781700	0.008617000	-4.351094800
O	-0.434576200	-0.967730300	-2.529254200
O	0.776053300	-0.064163800	2.244128400
H	1.674864600	-0.098243200	1.875311600
H	0.754727600	0.711337600	2.803885900
C	3.897265500	-1.971359200	0.111388300

C	-4.026860400	1.944730700	-0.084099700
H	4.862743500	-2.462717000	0.062647800
H	-4.994579000	2.429314500	-0.148995100

Table S26: Optimised geometry of the **La$^{(III)}$-isoamethyrin** complex with the BHLYP/def2-SVP model chemistry.

Atom	X	Y	Z
C	3.304849800	1.683759400	0.379944900
C	4.734555300	1.737546900	0.287878000
C	5.151586700	0.444346800	0.344477000
C	3.963216100	-0.361167900	0.465668400
N	2.863799800	0.419174800	0.498926400
H	5.335622000	2.628950800	0.178617200
H	6.163864400	0.067465700	0.292451300
C	2.362804800	2.729111600	0.348680000
C	2.542498200	4.165308000	0.329546500
N	1.070323800	2.417975900	0.369645300
C	1.293947400	4.700300100	0.356886100
H	3.486826700	4.690373300	0.307721800
C	0.384260900	3.580323500	0.386864400
H	1.021293300	5.746140400	0.359536000
C	-1.001625300	3.482490900	0.435040200
C	-2.064070700	4.459749000	0.416489300
N	-1.516850400	2.236924600	0.467168800
C	-3.223295300	3.751377700	0.416726900
H	-1.944505200	5.533596300	0.391788800
C	-2.838254800	2.356323200	0.434882400
H	-4.233086400	4.135552500	0.392284200
C	-3.607610400	1.181212600	0.372067800
C	-5.033016800	1.037480200	0.318493400
N	-2.982568700	-0.008546700	0.325819600

C	-5.258993300	-0.298578900	0.234412800
H	-5.756539300	1.839762100	0.338995800
H	-6.207696300	-0.814040100	0.174370400
C	2.865122100	-2.609589000	0.517725100
C	3.037909700	-4.043416200	0.551622200
N	1.537075000	-2.295251800	0.456493800
C	1.800424700	-4.583188400	0.502684300
H	3.990500600	-4.552590100	0.600384100
H	1.526288200	-5.627316100	0.502290800
C	0.895694400	-3.462931000	0.438353000
C	-3.964092300	-0.932256500	0.240957100
C	-2.549911000	-4.456917200	0.222239600
C	-1.244835400	-4.806874500	0.310923500
C	-2.591214500	-3.017746500	0.216223600
H	-3.415026400	-5.103642300	0.173631000
C	-0.512968800	-3.570514400	0.347503500
N	-1.324720600	-2.510065500	0.284984100
H	-0.821922000	-5.799377900	0.347313100
C	-3.778452800	-2.309898200	0.181216100
H	-4.680911200	-2.908283000	0.119631300
C	3.951512300	-1.753116600	0.514455500
H	4.923410600	-2.234727700	0.523729700
O	0.040646100	0.150293800	-4.088150200
O	-0.871337300	0.762443300	-2.249617900
O	0.740404000	-0.617099200	-2.214760800
N	-0.028429200	0.102359600	-2.907856600
La	-0.173604200	-0.049081700	0.072363100

O	0.814441000	0.191719500	2.473395800
H	1.709347500	0.214018500	2.088598300
H	0.698521400	1.042332000	2.896453000

Table S27: Optimised geometry of the **La$^{(III)}$-pent1** complex with the BHLYP/def2-SVP model chemistry.

Atom	X	Y	Z
C	0.0749831	-2.1336099	2.9771576
C	-0.3751855	-3.4144766	3.4744571
C	-0.5203765	-4.2119264	2.3981327
C	-0.1377598	-3.4222992	1.2492056
N	0.1769020	-2.1545634	1.6259588
H	-0.5417842	-3.6499053	4.5163558
H	-0.8290492	-5.2474078	2.3622126
C	0.4386171	-1.1167178	3.8506366
H	0.3997988	-1.4000358	4.8962849
C	0.8312205	0.2011684	3.6410506
C	1.2091385	1.0209488	4.7732509
N	0.8284313	0.9295203	2.4977960
C	1.3965113	2.2640776	4.2961667
H	1.2871356	0.6684986	5.7922976
C	1.0998261	2.2052510	2.8796954
H	1.6575247	3.1636296	4.8360154
C	0.9326473	3.3880277	2.1756801
H	1.1094505	4.2792168	2.7672223
C	0.4275410	3.6505002	0.9035494
C	-0.0178083	4.9738843	0.5452308
N	0.2569288	2.7912721	-0.1298958
C	-0.4991653	4.8881772	-0.7137794
H	0.0178217	5.8371240	1.1950923
C	-0.2814948	3.5293302	-1.1403223

H	-0.9312596	5.6684859	-1.3247144
C	-0.4518431	3.1581361	-2.4650240
C	0.0230154	-4.0063544	0.0048972
H	-0.1853302	-5.0699192	-0.0194562
H	-0.8568184	3.9374283	-3.1006115
C	-0.0427946	2.0142257	-3.1459596
C	0.0343462	2.0155798	-4.5849073
N	0.4316420	0.8480617	-2.6378401
C	0.6242233	0.8522195	-4.9338477
H	-0.2957967	2.8233541	-5.2230835
C	0.8224277	0.1161245	-3.7098727
H	0.8683741	0.4895158	-5.9225916
C	0.4995046	-3.4915381	-1.2003508
C	0.8562262	-4.3884500	-2.2768897
N	0.6966998	-2.2040479	-1.5582792
C	1.2946950	-3.6122935	-3.2862902
H	0.7783445	-5.4659486	-2.2369258
C	1.1359309	-2.2450802	-2.8416922
H	1.6450141	-3.9096332	-4.2648733
C	1.2453448	-1.2107070	-3.7589873
H	1.5738643	-1.5282034	-4.7422873
La	0.3195028	0.0142624	-0.0316823
N	-2.6636983	-0.1353516	0.1858622
O	-2.0303645	-0.6804378	-0.7542327
O	-1.9554964	0.4982120	1.0092025
O	-3.8415725	-0.2141807	0.2886978
O	2.6223316	-1.0510391	0.6991944

H	3.1716522	-0.4181916	1.1612337
O	2.6241069	1.3696301	-0.5529197
H	2.8922364	1.2127065	-1.4585230
H	2.3195481	-1.7029763	1.3356852
H	2.1011708	2.1823540	-0.5751238

Table S28: Optimised geometry of the **La$^{(III)}$-rosarin** complex with the BHLYP/def2-SVP model chemistry.

Atom	X	Y	Z
C	2.326861900	3.157475300	-0.439859300
C	2.614683100	4.432917400	-0.986071100
C	1.473862200	5.180942100	-0.804566900
C	0.530736000	4.324848100	-0.185202300
N	1.082313300	3.091751700	0.024868900
H	3.542468100	4.740742200	-1.447619600
H	1.299694200	6.208201900	-1.098104700
C	-0.780830700	4.639515100	0.150853500
H	-1.047890900	5.691553200	0.100583400
C	-1.815323000	3.779576500	0.462181300
C	-3.142977800	4.254730900	0.692762700
N	-1.810943700	2.396219100	0.381079900
C	-3.950836500	3.159720400	0.715947300
H	-3.422979100	5.295550600	0.793304200
C	-3.082508700	2.045698600	0.490039400
H	-5.023017200	3.122949100	0.843623300
C	-3.540437000	0.694526900	0.282642200
C	-4.900884300	0.341846700	0.104982300
N	-2.720658800	-0.359291800	0.168678500
C	-4.903383500	-1.002977900	-0.151146600
H	-5.744413500	1.015641600	0.136221800
C	-3.553391300	-1.426556400	-0.094249400
H	-5.751683200	-1.645720000	-0.347510600
C	3.241589400	2.062544600	-0.244666800

C	4.664322600	2.204292100	-0.323361100
N	2.863329200	0.850558900	0.141599800
C	5.171098000	1.013207700	0.085081100
H	5.196212300	3.099440500	-0.612334000
C	4.049373700	0.167837600	0.362247300
H	6.208818200	0.720910800	0.181898200
C	4.208441200	-1.133200600	0.759837400
C	-3.193990200	-2.762807400	-0.200202500
H	5.237843000	-1.435141000	0.930259400
H	-4.010661900	-3.444560400	-0.417913900
C	3.273090500	-2.159802800	0.913095000
C	3.646393500	-3.490578900	1.211568700
N	1.928837400	-2.082801900	0.657723700
C	2.503611700	-4.250918000	1.110334600
H	4.650428100	-3.823102100	1.441396600
C	1.482848100	-3.344381000	0.756922500
H	2.395377700	-5.317485500	1.248573300
C	-1.964957900	-3.350348600	-0.010708700
C	-1.751413800	-4.756325100	-0.017706100
N	-0.787818500	-2.693106900	0.288151800
C	-0.436764400	-4.955588400	0.297893700
H	-2.508911200	-5.500327600	-0.227995900
C	0.113587300	-3.656572700	0.467249000
H	0.098829100	-5.890272200	0.382897200
O	0.724101800	1.767127400	2.202790000
H	1.568486100	1.674064100	2.654008000
H	0.865170400	2.409776400	1.396558100

O	0.520375900	-1.188464500	2.915252300
H	0.966444500	-2.043110800	2.903607900
H	-0.226666700	-1.191424900	3.524459200
La	-0.012513300	-0.132102200	0.643460600
O	-1.482807200	0.455959800	2.772899800
H	-1.023551100	1.296411800	2.940200700
H	-2.397195300	0.648732300	2.536491500

Table S29: Optimised geometry of the **La$^{(III)}$-rubyrin** complex with the BHLYP/def2-SVP model chemistry.

Atom	X	Y	Z
C	2.018998600	3.906673200	0.408649300
C	2.165249800	5.205921200	-0.167124700
C	1.052649700	5.391060700	-0.941130900
C	0.252024000	4.220434800	-0.771725400
N	0.865266900	3.342962500	0.059525900
H	3.010846600	5.868982000	-0.039901700
H	0.805000000	6.243523300	-1.560407000
C	-1.005493100	4.036373300	-1.345061600
H	-1.335525600	4.864835100	-1.966179500
C	-1.960861200	3.045235600	-1.183558700
C	-3.319872800	3.326642600	-1.538850100
N	-1.888843900	1.863548800	-0.472825400
C	-4.082307700	2.360360900	-0.956479600
H	-3.649742500	4.187038000	-2.106414200
C	-3.152384000	1.486108700	-0.323076000
H	-5.157347300	2.249482700	-0.974668800
C	3.054747800	3.233043400	1.126506300
H	3.813313400	3.874975500	1.566236600
C	-3.525949700	0.300457200	0.408714300
C	-4.818833600	0.047042200	0.999485200
N	-2.695146900	-0.697133900	0.596537200
C	-4.726528700	-1.173914200	1.576559700
H	-5.664808100	0.719157900	0.982281700
C	-3.405176000	-1.671578900	1.277572800

H	-5.491082700	-1.720123500	2.113141900
C	3.330383700	1.900110200	1.152976400
C	4.640479200	1.414140500	1.543764700
N	2.632384200	0.878051700	0.509371900
C	4.756323900	0.168535800	1.039386900
H	5.383707700	1.994437400	2.074925100
C	3.484304700	-0.108005400	0.409004700
H	5.599785400	-0.505399500	1.090421400
C	-3.018582200	-2.964897400	1.403172300
H	-3.712513900	-3.629865900	1.907524600
C	3.164667100	-1.333640200	-0.290956100
C	4.104250600	-2.197653100	-0.917459600
N	1.919129600	-1.777958200	-0.398850700
C	3.367313400	-3.225490700	-1.434812100
H	5.173250800	-2.047212200	-0.969542700
C	2.011677300	-2.980583100	-1.063259400
H	3.721190700	-4.095857200	-1.971778500
C	-1.912175700	-3.609251200	0.771905700
C	-1.951664200	-5.007291500	0.505440200
N	-0.842427700	-3.021509900	0.208728100
C	-0.886796300	-5.262517300	-0.309894000
H	-2.708425700	-5.694643900	0.859052100
C	-0.197192200	-4.026072300	-0.456829900
H	-0.582199500	-6.206636400	-0.742024500
C	1.039497300	-3.958193600	-1.102322700
H	1.328398700	-4.869312300	-1.617981600
N	0.325406200	-1.205385200	3.474544100

O	-0.793855500	-1.239967700	2.905951000
O	1.271507900	-0.796830100	2.751727900
O	0.478416100	-1.524690500	4.604108400
La	-0.085087200	-0.369542200	0.620410400
O	-0.013551700	1.734637600	1.973624800
H	0.629442500	1.798264900	2.685988200
H	0.219587200	2.426077700	1.275117400

Table S30: Optimised geometry of the **La**$^{(III)}$**-hex1** complex with the BHLYP/def2-SVP model chemistry.

Atom	X	Y	Z
C	1.7845954	3.2687164	-0.1155638
C	2.0007967	4.4931146	-0.8714398
C	1.3057361	4.3626002	-2.0141122
C	0.5398319	3.1283440	-1.8737147
N	0.9033241	2.4575246	-0.7748436
H	2.6403423	5.3082258	-0.5614253
H	1.2253673	5.0570738	-2.8389345
C	-0.6160857	2.9274910	-2.6439727
H	-0.7207521	3.5651440	-3.5143620
C	-1.8107500	2.4458735	-2.0973261
C	-3.0797513	3.0595767	-2.4795190
N	-1.9518608	1.6939877	-0.9946592
C	-3.9226081	2.8155575	-1.4636972
H	-3.2371605	3.6712679	-3.3570539
C	-3.2188683	1.9333510	-0.5431038
H	-4.9377221	3.1641483	-1.3314423
C	-3.8039332	1.4577500	0.6037714
C	2.4584469	2.9990869	1.0481298
H	-4.7358816	1.9264283	0.8985635
H	2.9764892	3.8312901	1.5107233
C	-3.4446169	0.3012012	1.3249207
C	-4.2386522	-0.2120890	2.4289152
N	-2.5454756	-0.5907771	0.9205137
C	-3.8414550	-1.4856769	2.6142813

H	-5.0181464	0.3328963	2.9429957
C	-2.8317431	-1.7424058	1.6031771
H	-4.2259927	-2.2176229	3.3110022
C	2.7667342	1.7217792	1.5619073
C	3.7452328	1.5299205	2.6176647
N	2.4668155	0.5721037	0.9637562
C	4.1036578	0.2321639	2.5612155
H	4.1285177	2.3085602	3.2623624
C	3.3416852	-0.3486402	1.4728025
H	4.8467312	-0.2885313	3.1493042
C	3.7029128	-1.5062404	0.8378434
C	-2.5286050	-2.9980655	1.1534221
H	4.5699070	-2.0242510	1.2339173
H	-2.9965332	-3.8281287	1.6715296
C	3.1717377	-2.0290016	-0.3639779
C	3.9206473	-2.8876222	-1.2369623
N	1.9283646	-1.8290210	-0.8268431
C	3.0942138	-3.1884497	-2.2729754
H	4.9412761	-3.2078054	-1.0790942
C	1.8262080	-2.5921733	-1.9488211
H	3.2899524	-3.8296060	-3.1217097
C	-1.8493908	-3.3161851	-0.0485391
C	-2.1467224	-4.4955828	-0.8151632
N	-0.9167553	-2.5674471	-0.6486640
C	-1.3946946	-4.4103244	-1.9423699
H	-2.8537481	-5.2647228	-0.5367001
C	-0.5668592	-3.2447530	-1.7763846

H	-1.3366629	-5.1110568	-2.7640418
C	0.6406811	-3.1321414	-2.4560608
H	0.7500612	-3.7662599	-3.3300573
La	-0.0339780	-0.0337641	-0.0168300
N	-0.0904146	0.1463490	2.9829531
O	-0.1218912	0.2159755	4.1689312
O	-0.3181563	1.1384984	2.2488697
O	0.1710846	-0.9242741	2.3828492
O	0.1354304	-0.0283358	-2.6459498
H	0.8460021	-0.5014221	-3.0788639
H	0.0164929	0.8170801	-3.0895694

Gd-Complexes

Table S31: Optimised geometry of the **Gd**$^{(III)}$**-pent0** complex with the BHLYP/def2-SVP model chemistry.

Atom	X	Y	Z
C	-2.7449727	-0.9817903	-0.5396616
C	-3.7991232	-0.5160146	0.2981516
C	-3.7358402	0.8640150	0.2942648
C	-2.6447485	1.2281902	-0.5470133
N	-2.1522449	0.0978326	-1.1180527
H	-4.4616909	-1.1285202	0.8945084
H	-4.3394298	1.5372335	0.8878186
C	-1.9523368	-2.1742348	-0.5295228
C	-1.9716554	-3.3419418	0.2944502
N	-0.7125666	-2.0443397	-1.0569521
C	-0.6835319	-3.8307385	0.3228924
H	-2.8185771	-3.7209066	0.8502432
C	0.1085624	-2.9572397	-0.4843920
H	-0.3233689	-4.6678713	0.9053894
C	-1.7449737	2.3417060	-0.5328599
C	-1.6470961	3.4928439	0.3065621
N	-0.5237307	2.1012519	-1.0695213
C	-0.3161742	3.8532741	0.3381131
H	-2.4509242	3.9430629	0.8730540
C	0.3860112	2.9185778	-0.4816180
H	0.1246211	4.6408960	0.9340568
C	1.7210279	2.4061328	-0.4409838

C	2.8351310	2.6640088	0.4185670
N	1.8796965	1.1780124	-0.9848070
C	3.5918209	1.5133761	0.4468456
H	3.0062852	3.5636115	0.9939916
C	2.9329867	0.5639290	-0.3959363
H	4.4724164	1.3343405	1.0487233
C	1.4893320	-2.5865953	-0.4407924
C	2.5546091	-2.9327283	0.4458439
N	1.7813048	-1.3901825	-1.0080245
C	3.4179259	-1.8575345	0.4746170
H	2.6235224	-3.8306601	1.0448053
C	2.8725625	-0.8640822	-0.3945298
H	4.2936710	-1.7506770	1.1003041
Gd	0.0933646	-0.0101307	-1.9892129
O	0.0553778	1.5970678	-3.9736139
H	-0.7300809	1.2802114	-4.4272870
O	-1.7630961	-0.4873899	-3.8119188
H	-1.8687072	-1.3979777	-4.0866071
O	1.5296823	-0.9651558	-3.8246065
H	2.0264450	-0.3382166	-4.3499724
H	-0.1989903	2.4029502	-3.5166967
H	2.1499337	-1.3888356	-3.2204774
H	-2.3657533	-0.3588352	-3.0620717

Table S32: Optimised geometry of the **Gd$^{(III)}$-porph1212** complex with the BHLYP/def2-SVP model chemistry.

Atom	X	Y	Z
C	3.004051700	1.439355900	0.199659800
C	4.297281800	1.126404200	-0.310278900
C	4.236051900	-0.170854500	-0.728255400
C	2.925031400	-0.635326400	-0.424863200
N	2.184815600	0.393443700	0.131016800
H	5.135067000	1.808764200	-0.355969300
H	5.020254300	-0.767383600	-1.176000400
C	0.421229700	3.333122500	-0.199785800
C	-0.138401700	4.361963400	-1.004888300
N	-0.318483000	2.222041600	-0.242466900
C	-1.256871700	3.827117200	-1.583739200
H	0.274897800	5.353166400	-1.133442500
C	-1.377082500	2.502432200	-1.086911800
H	-1.945997900	4.301450700	-2.270541300
C	1.426109100	-2.680151400	-0.170575000
C	1.377985500	-4.092824700	-0.249280600
N	0.272684600	-2.197718800	0.407949000
C	0.194026400	-4.475481200	0.324866000
H	2.153114100	-4.722719000	-0.665845700
C	-0.469806400	-3.278766600	0.690972200
H	-0.195045800	-5.476361600	0.455084300
C	-2.905040600	0.478844300	-0.843955300
C	-4.199498500	-0.036431400	-1.120930700
N	-2.238663900	-0.361735900	0.027728500

C	-4.331245500	-1.176799800	-0.380846200
H	-4.925692200	0.421663200	-1.779812500
C	-3.090613200	-1.355754900	0.291054500
H	-5.179105300	-1.845807500	-0.322741100
C	2.558127600	-1.959642600	-0.554561600
H	3.329533600	-2.581128300	-1.000190700
C	-2.877476000	-2.536555000	1.114071200
C	-1.802580700	-3.330878600	1.267652700
H	-3.797263100	-2.907172600	1.562013900
H	-1.982028000	-4.247807500	1.825898800
C	1.640703400	3.548456300	0.568569900
C	2.708172700	2.761785400	0.736625300
H	1.722323400	4.553822100	0.976739200
H	3.546587100	3.204181700	1.270158800
C	-2.485401300	1.706068700	-1.337884600
H	-3.197171600	2.160855200	-2.021073900
O	-1.046820000	-0.991541500	3.095341300
H	-0.288459100	-1.478297500	3.446868400
H	-1.744165100	-1.628173500	2.886198300
O	-1.627949600	1.716759100	2.314422300
H	-1.128938500	1.933697700	3.113121500
H	-1.729380800	2.528432400	1.806275500
N	1.680198300	0.427165900	3.493145400
O	1.564373900	-0.687987300	2.939584600
O	0.948347500	1.339574100	3.017527300
O	2.416964200	0.624605400	4.400209000
Gd	-0.051843300	0.152065600	1.135764200

Table S33: Optimised geometry of the **Gd$^{(III)}$-porph1122** complex with the BHLYP/def2-SVP model chemistry.

Atom	X	Y	Z
C	2.863206000	1.627905400	0.093572200
C	3.990895200	1.698694500	-0.759035700
C	4.186926900	0.426233700	-1.222191500
C	3.185616300	-0.393264500	-0.635032900
N	2.351364000	0.397566700	0.144320000
H	4.577319300	2.585631100	-0.956111200
H	4.976715900	0.069566700	-1.870772700
C	0.173173700	3.315633200	0.138570300
C	-0.517995500	4.376705600	-0.505873200
N	-0.464088400	2.153509200	0.001315900
C	-1.630240300	3.813619700	-1.070129500
H	-0.199466900	5.409861000	-0.535855700
C	-1.605924300	2.432653400	-0.731161900
H	-2.406255300	4.300741900	-1.646572900
C	1.453174500	-3.159777500	0.403980300
C	1.121598700	-4.535330200	0.779040500
N	0.363365000	-2.377110600	0.495872600
C	-0.192073700	-4.568687900	1.032727800
H	1.833130000	-5.349871200	0.797582700
C	-0.693633600	-3.223696400	0.788122100
H	-0.807945300	-5.416468100	1.302974600
C	-2.882470300	0.253992000	-0.603960000
C	-4.103627400	-0.437521700	-0.838379200
N	-1.993307200	-0.569599100	0.043608100

C	-3.950612700	-1.689849600	-0.312360700
H	-4.969786500	-0.024993400	-1.339463400
C	-2.620980200	-1.743355100	0.196701200
H	-4.659360700	-2.507092000	-0.309218400
C	2.719676800	-2.897602900	-0.124695900
C	3.327220000	-1.780298500	-0.676308100
H	3.315818100	-3.799327900	-0.223205700
H	4.220294000	-2.048111900	-1.237022800
C	-2.023026900	-2.941565200	0.703755900
H	-2.703702900	-3.765855800	0.890306000
C	1.378235200	3.505738500	0.941014900
C	2.482337100	2.765421700	0.950168100
H	1.362343400	4.381274500	1.588040000
H	3.276800000	3.082368100	1.624995000
C	-2.666941900	1.574585000	-0.976976900
H	-3.490107200	2.026178700	-1.523324900
O	-1.601324700	1.433435600	2.568850800
H	-0.929749600	1.750050600	3.192291600
H	-1.879541900	2.174320500	2.020721600
O	-1.177389300	-1.079861400	2.998166300
H	-1.469577100	-1.983402400	2.828808700
H	-1.951997600	-0.506011700	3.060419900
Gd	0.153721900	-0.020810700	1.173050200
N	1.619757900	0.078571800	3.654439500
O	1.683053800	-0.912955700	2.910004500
O	0.934969700	1.047504100	3.199350900
O	2.151367500	0.140683200	4.711808200

Table S34: Optimised geometry of the **Gd$^{(III)}$-sapphyrin** complex with the BHLYP/def2-SVP model chemistry.

Atom	X	Y	Z
C	-0.024831800	-2.459238700	2.899606900
C	0.045114500	-3.874327900	3.071360600
C	0.299225900	-4.409936700	1.840026000
C	0.359664000	-3.308302100	0.944096700
N	0.174685200	-2.139260600	1.590295400
H	-0.079735400	-4.395121300	4.011752200
H	0.413697700	-5.452175600	1.576356700
C	-0.234024700	-1.544285000	3.919014800
H	-0.383844100	-1.955521100	4.911667800
C	-0.203763200	-0.163438000	3.835993500
C	-0.244486500	0.650433800	5.013059600
N	-0.017381500	0.616537200	2.718340500
C	-0.029304700	1.926141800	4.612074900
H	-0.392417500	0.275324200	6.017276300
C	0.098365600	1.887347700	3.183405300
H	0.024762300	2.823693700	5.213829500
C	0.299461400	3.066043200	2.457429900
H	0.418370800	3.947779100	3.077855300
C	0.242899300	3.333618600	1.102854500
C	0.251292600	4.702475900	0.649640300
N	0.062235300	2.476865300	0.048972100
C	0.010651000	4.671630200	-0.674444000
H	0.386170500	5.561971100	1.292949800
C	-0.097766400	3.282505500	-1.050673600

H	-0.084185300	5.500604100	-1.363317800
C	-0.331481200	2.967268600	-2.377930700
H	-0.482877900	3.830429600	-3.017098700
C	-0.341655000	1.748292400	-3.055790000
C	-0.525962100	1.664524100	-4.473259900
N	-0.103505100	0.515245300	-2.527680200
C	-0.358102900	0.359345700	-4.811138800
H	-0.735132800	2.504572900	-5.122408800
C	-0.089392800	-0.351656500	-3.600703200
H	-0.407870300	-0.090037600	-5.794530300
C	0.490326800	-3.321686000	-0.457919700
C	0.638829800	-4.444712900	-1.316888500
N	0.383128400	-2.171276600	-1.155209300
C	0.592129500	-3.946771400	-2.586664800
H	0.744048500	-5.474729900	-1.005730800
C	0.409874400	-2.533067300	-2.474094500
H	0.647064900	-4.493052100	-3.519399900
C	0.185242200	-1.708024500	-3.570431600
H	0.197387300	-2.202617500	-4.536258800
O	2.292202800	0.115720400	-1.300130400
H	2.563574700	-0.773846000	-1.555787400
H	1.637429900	0.394908800	-1.983216200
O	2.435502300	0.679742000	1.252256200
H	2.977788100	1.091536000	0.569640800
H	2.334854700	1.277393900	2.002830500
Gd	0.326887000	-0.074770000	0.285642200
O	-1.965659000	0.211016800	-0.426539000

H	-2.340850600	1.057583800	-0.157596300
H	-1.724155100	0.291160600	-1.372708800

Table S35: Optimised geometry of the **Gd$^{(III)}$-porph2** complex with the BHLYP/def2-SVP model chemistry.

Atom	X	Y	Z
C	2.390589170	0.646157650	-0.695891750
C	3.927858600	0.597725100	-0.680128850
C	4.300855620	0.067126810	0.468984950
C	3.022446370	-0.395845390	1.142962510
N	1.937313340	0.082923730	0.467076120
H	4.577539430	0.944967540	-1.455962550
H	5.300497670	-0.038912990	0.835583010
C	-0.203059070	2.413232050	-0.892117520
C	-0.809915860	3.823672070	-1.019896600
N	-0.725997110	1.837274850	0.240018150
C	-1.648871100	3.991957060	-0.014204990
H	-0.587409410	4.541150610	-1.782635330
C	-1.714022570	2.650837010	0.691255990
H	-2.191259040	4.880555310	0.233293630
C	1.609197690	-2.822084480	1.165447040
C	1.785942640	-4.187872880	0.523666680
N	0.644768370	-2.135697850	0.497196730
C	1.075809320	-4.162431810	-0.594002400
H	2.380923140	-4.999252450	0.890080050
C	0.321264050	-2.820863900	-0.644231700
H	1.027342010	-4.940002490	-1.326612050
C	-3.271856300	0.336157960	0.422816050
C	-4.399556860	-0.084550170	-0.506284250
N	-2.122220730	-0.305649450	0.054726700

C	-3.842347650	-0.813607240	-1.458413350
H	-5.434677520	0.173306390	-0.418931650
C	-2.358690730	-1.001728440	-1.101296880
H	-4.338262230	-1.212635950	-2.318287980
C	2.401446890	-2.424394750	2.263320720
C	3.080535240	-1.240398790	2.261105090
H	2.535111710	-3.097453030	3.083611320
H	3.722749370	-0.999695270	3.082628390
C	-1.613937110	-1.828892790	-1.960169530
C	-0.507759020	-2.601724910	-1.761590910
H	-2.014966810	-1.898419840	-2.949407990
H	-0.235554890	-3.182956460	-2.617035180
C	0.714503410	2.035762250	-1.879210930
C	1.813195030	1.259159350	-1.805517720
H	0.539808070	2.468711810	-2.841936910
H	2.349464580	1.141898530	-2.723815010
C	-3.501529130	1.272461240	1.457700450
C	-2.726508160	2.383215920	1.614821110
H	-4.352466740	1.146117740	2.092491650
H	-2.951759000	3.084711810	2.391074380
N	1.468460320	0.686977870	3.610944180
O	0.939568110	-0.434583680	3.493190830
O	1.065860300	1.398637630	2.672156830
O	-2.058481540	0.553269970	2.757485050
H	-1.781669770	0.684806840	3.666566700
H	-2.397887550	1.378907630	2.402404870
O	2.326677830	1.069339810	4.593654200

O	-1.293305430	-2.109090840	2.218594930
H	-0.782907110	-2.503866110	2.928925370
H	-2.171275380	-1.890356680	2.539376100
Gd	-0.222335690	-0.153616730	1.475149010

Table S36: Optimised geometry of the **Gd$^{(III)}$-amethyrin** complex with the BHLYP/def2-SVP model chemistry.

Atom	X	Y	Z
C	2.887816400	-2.802674400	0.054079900
C	2.968203200	-4.254439100	0.107009600
C	1.711900400	-4.694503900	0.327310600
C	0.896924400	-3.497784500	0.381229500
N	1.614902200	-2.389185900	0.211195200
H	3.874727200	-4.834903900	0.002945100
H	1.365099600	-5.711789600	0.437341000
C	3.977964000	-0.595418800	0.054737100
C	5.180333600	0.221237300	0.117234600
N	2.876408200	0.164082400	0.215670700
C	4.767439100	1.484307600	0.351624700
H	6.192000900	-0.146024600	0.011428600
C	3.321934000	1.405124300	0.403705900
H	5.365492200	2.375794100	0.473533400
C	2.411028500	2.447636700	0.579115800
C	2.673987900	3.851895900	0.709975200
N	1.087992100	2.208233600	0.522501200
C	1.459993800	4.464996300	0.690638800
H	3.649426600	4.310640400	0.782396500
C	0.496847500	3.413048800	0.549708700
H	1.246229000	5.522632000	0.743984200
C	-0.879578600	3.495910500	0.333993400
C	-1.694631700	4.673089100	0.239396200
N	-1.587351000	2.372502200	0.130071000

C	-2.942029000	4.225315600	-0.039241400
H	-1.357896300	5.691847400	0.364363400
H	-3.845111600	4.801880100	-0.184675300
C	-0.487917500	-3.409419500	0.530170600
C	-1.445669800	-4.473665500	0.621624600
N	-1.101121700	-2.213017000	0.470468400
C	-2.669847600	-3.882815900	0.572479400
H	-1.218400400	-5.527741500	0.688169800
H	-3.640359500	-4.357038100	0.591109000
C	-2.417053600	-2.476996200	0.455855100
C	-2.861160600	2.786429400	-0.089070700
C	-5.138765800	-0.225632400	-0.143927800
C	-4.741720800	-1.495899900	0.107407400
C	-3.946703200	0.585582200	-0.145237900
H	-6.142520600	0.145652500	-0.298019000
C	-3.314003400	-1.430972500	0.233743000
N	-2.847799600	-0.181625900	0.070619100
H	-5.348556300	-2.385092000	0.196613900
N	-0.058125200	0.060940400	-2.898470900
O	0.333694800	1.036143400	-2.202183000
O	0.012615000	0.040001800	-4.074845100
O	-0.533693500	-0.888435200	-2.219610300
C	3.994987000	-1.976733000	-0.063693800
C	-3.959848700	1.963816900	-0.261736200
H	4.960905900	-2.453715000	-0.186129200
H	-4.918072900	2.440765400	-0.431123100
Gd	-0.230914700	0.116018200	-0.095486900

Table S37: Optimised geometry of the **Gd$^{(III)}$-isoamethyrin** complex with the BHLYP/def2-SVP model chemistry.

Atom	X	Y	Z
C	3.808805300	-0.289083000	-0.067433400
C	5.068803700	-0.988193200	-0.100849800
C	4.765776200	-2.276043200	0.188812500
C	3.329485800	-2.317752900	0.372818000
N	2.785805900	-1.105367900	0.206132100
H	6.037309100	-0.556284900	-0.308839100
H	5.435534500	-3.121668100	0.265599800
C	3.547999300	1.080365900	-0.146919500
C	4.458661500	2.200086200	-0.209017800
N	2.291650500	1.495822200	0.029402400
C	3.702524200	3.308667000	-0.005491800
H	5.527538300	2.142122500	-0.357045700
C	2.352894600	2.834410100	0.164905300
H	4.027776000	4.338139700	0.042612000
C	1.168172000	3.467905500	0.505748200
C	0.812643900	4.841715700	0.720925400
N	0.084453200	2.671030700	0.617629500
C	-0.532150600	4.847960800	0.932649000
H	1.485316600	5.687181800	0.695648700
C	-0.953143100	3.475399400	0.834376100
H	-1.171804000	5.700261700	1.111608400
C	-2.233712300	2.908153100	0.768566400
C	-3.512373000	3.556437900	0.797307400
N	-2.330307800	1.602377900	0.488382900

C	-4.409022800	2.588784900	0.480963800
H	-3.694791700	4.600827600	1.005742700
H	-5.482384800	2.674898600	0.381823800
C	1.224293200	-3.577087500	0.694912300
C	0.596029400	-4.874771400	0.783623900
N	0.285559100	-2.602456800	0.488533900
C	-0.720990300	-4.679940200	0.562660600
H	1.119611700	-5.805046400	0.955449300
H	-1.511623300	-5.413880900	0.525339000
C	-0.867138000	-3.258777700	0.380213500
C	-3.648561400	1.379907400	0.288857200
C	-4.241654000	-2.286435200	-0.304423200
C	-3.333314100	-3.275325000	-0.126479500
C	-3.532904800	-1.047411100	-0.147536700
H	-5.302881500	-2.373705100	-0.491680500
C	-2.085618900	-2.610508100	0.103609200
N	-2.200566800	-1.277465800	0.061921600
H	-3.492503600	-4.342677600	-0.148855600
C	-4.199034900	0.160692000	-0.063303100
H	-5.269781700	0.133922500	-0.230901700
Gd	0.020937000	0.184222500	0.004664200
C	2.598744000	-3.462758100	0.665902400
H	3.158572300	-4.379348300	0.815366500
O	-0.003662200	0.294361500	-3.969687100
O	-0.525587800	1.148801400	-2.072339200
O	0.521445000	-0.683774500	-2.134465400
N	-0.002340000	0.254145500	-2.791641400

Table S38: Optimised geometry of the **Gd$^{(III)}$-pent1** complex with the BHLYP/def2-SVP model chemistry.

Atom	X	Y	Z
C	-0.4693038	-2.0626368	2.8662386
C	-1.1207639	-3.2664937	3.3325225
C	-1.6070908	-3.8839821	2.2401891
C	-1.1807840	-3.0908736	1.1068593
N	-0.5718300	-1.9415563	1.5240139
H	-1.1727995	-3.5796577	4.3659365
H	-2.1344234	-4.8255003	2.1735524
C	0.2234564	-1.2379238	3.7453935
H	0.3493174	-1.6276094	4.7485644
C	0.7089703	0.0379966	3.5334525
C	1.3534078	0.7864401	4.5926200
N	0.4976764	0.8225157	2.4481335
C	1.4653510	2.0510698	4.1493365
H	1.6386362	0.3773856	5.5517854
C	0.8733410	2.0726768	2.8278242
H	1.8609466	2.9162987	4.6627378
C	0.5409503	3.2590807	2.2102605
H	0.7954008	4.1617740	2.7529104
C	-0.2081343	3.4449162	1.0491444
C	-0.8108862	4.7224456	0.7468868
N	-0.4606274	2.5430348	0.0791956
C	-1.4491020	4.5704548	-0.4296574
H	-0.7433499	5.6022710	1.3714005
C	-1.1483826	3.2292694	-0.8789078

H	-2.0070659	5.3032321	-0.9959277
C	-1.2733066	2.9110235	-2.2153803
C	-1.1470717	-3.6535828	-0.1535718
H	-1.5918789	-4.6390743	-0.2329332
H	-1.7694865	3.6557033	-2.8274595
C	-0.6007134	1.9087484	-2.9198085
C	-0.2804742	2.0590430	-4.3128023
N	-0.0383492	0.7908596	-2.4102684
C	0.5667027	1.0461119	-4.6167353
H	-0.6227613	2.8638193	-4.9484390
C	0.6547597	0.2304571	-3.4375584
H	1.0455997	0.8299460	-5.5616927
C	-0.4039598	-3.2421069	-1.2651135
C	0.0029471	-4.1690599	-2.2890519
N	0.0891963	-2.0112755	-1.5019377
C	0.8108342	-3.4770349	-3.1259559
H	-0.2716464	-5.2140111	-2.3294151
C	0.7906672	-2.1147064	-2.6617977
H	1.3198291	-3.8269569	-4.0133454
C	1.1596065	-1.0639603	-3.4882803
H	1.7026331	-1.3292007	-4.3881641
Gd	0.2132774	0.0514002	0.0595411
N	3.0555063	0.0374021	0.1630482
O	4.2350006	0.0551338	0.2578029
O	2.3932782	1.0209863	-0.2596604
O	2.3627105	-0.9631735	0.4768386
O	-2.1787731	-0.2603953	-0.5355849

H	-2.1293663	-0.2441926	-1.4952416
H	-2.4853052	-1.1376594	-0.2986857

Table S39: Optimised geometry of the **Gd$^{(III)}$-rosarin** complex with the BHLYP/def2-SVP model chemistry.

Atom	X	Y	Z
C	1.978787300	2.693912000	-0.828247600
C	2.281742600	4.075252500	-0.957724100
C	1.303629000	4.729280100	-0.251461300
C	0.416537400	3.729998000	0.231548100
N	0.845481700	2.486497200	-0.161449600
H	3.143426200	4.500214100	-1.453521900
H	1.200102900	5.794760000	-0.089119200
C	-0.732431600	3.968079800	0.975976900
H	-0.889741300	4.986220600	1.320474600
C	-1.767693500	3.071338500	1.174295500
C	-3.104810000	3.481840500	1.466121100
N	-1.778555900	1.808560600	0.610624700
C	-3.926093700	2.503101100	0.978577300
H	-3.387674400	4.428916400	1.908142300
C	-3.057042700	1.510123800	0.443765400
H	-5.006938100	2.477801600	0.970932100
C	-3.398406500	0.324993200	-0.294500000
C	-4.643121900	-0.037297900	-0.861641800
N	-2.454322800	-0.589322200	-0.509355200
C	-4.435784400	-1.266439600	-1.442086900
H	-5.556571700	0.540266600	-0.827715100
C	-3.087917500	-1.618351900	-1.164406400
H	-5.159744200	-1.895401100	-1.944371300
C	2.892753200	1.588782800	-0.978436400

C	4.246897400	1.650988900	-1.404680100
N	2.616806800	0.462719100	-0.324834800
C	4.842465900	0.534483000	-0.882752000
H	4.706983600	2.457603200	-1.958528800
C	3.829539700	-0.178709600	-0.182395000
H	5.885547000	0.249142300	-0.935596100
C	4.069260700	-1.169644200	0.750479000
C	-2.638106800	-2.933855400	-1.119458600
H	5.106939100	-1.447166600	0.911456200
H	-3.266747200	-3.686156500	-1.586574500
C	3.115582200	-1.874215300	1.473453400
C	3.395634600	-3.018133700	2.267095400
N	1.772355500	-1.824599300	1.177619900
C	2.216686400	-3.712528300	2.385238600
H	4.373756700	-3.301944200	2.634645600
C	1.265786800	-2.963631700	1.649995100
H	2.048056100	-4.664959200	2.868479600
C	-1.590356000	-3.391191000	-0.345715800
C	-1.445445000	-4.743196000	0.078186200
N	-0.657409100	-2.597675500	0.288068100
C	-0.447614300	-4.756015000	1.014587500
H	-2.050307000	-5.573767400	-0.262790900
C	0.025439300	-3.417268800	1.078647000
H	-0.054599500	-5.600596700	1.563015400
O	-0.242404900	0.455127700	-2.129146300
H	-0.010014200	1.387891000	-2.238507300
H	-1.197401500	0.359008700	-2.256718100

O	0.106684500	0.571631600	2.492587000
H	0.874938600	0.477080100	3.065390800
H	-0.315587500	1.429687100	2.638082000
Gd	0.108134500	-0.102636800	0.195112700

Table S40: Optimised geometry of the **Gd$^{(III)}$-rubyrin** complex with the BHLYP/def2-SVP model chemistry.

Atom	X	Y	Z
C	1.884177200	3.333880600	0.656735000
C	2.020402100	4.691520500	0.236801800
C	1.212141200	4.826984200	-0.855339000
C	0.544702000	3.574518400	-1.002569300
N	0.981669500	2.670880200	-0.084029200
H	2.673464800	5.429130500	0.684420800
H	1.046893200	5.706759700	-1.463545900
C	-0.581618900	3.441390800	-1.823608900
H	-0.743393500	4.244744500	-2.536460600
C	-1.630466400	2.560808500	-1.646069600
C	-2.975142500	2.887839300	-2.007972700
N	-1.633155800	1.563767700	-0.702203000
C	-3.787035000	2.156363700	-1.182809500
H	-3.265934200	3.638322600	-2.731627800
C	-2.908090600	1.363750200	-0.397123700
H	-4.866435000	2.169402200	-1.122760700
C	2.821346500	2.766888400	1.572364000
H	3.409461700	3.472556900	2.150973100
C	-3.203168700	0.408164600	0.642900800
C	-4.395924400	0.201779600	1.420394200
N	-2.266330300	-0.457206400	0.937185400
C	-4.132787100	-0.871968900	2.212452200
H	-5.303760800	0.786376800	1.366463400
C	-2.818337100	-1.340182100	1.837900100

H	-4.795729900	-1.357026100	2.917023100
C	3.232095600	1.468907200	1.549477400
C	4.570066200	1.021130600	1.860257200
N	2.621897700	0.535614200	0.741759400
C	4.781023000	-0.097617200	1.116320700
H	5.278464000	1.545584100	2.488199200
C	3.538542300	-0.347127300	0.435422500
H	5.684379400	-0.685958500	1.034940300
C	-2.404031300	-2.635260700	1.909067100
H	-2.949618900	-3.304675300	2.566967700
C	3.175550600	-1.361476900	-0.524295300
C	4.000776300	-2.200870200	-1.319581000
N	1.883284400	-1.576501800	-0.729971000
C	3.136261000	-2.978096700	-2.043214500
H	5.081792400	-2.212644100	-1.332107500
C	1.818569100	-2.627474300	-1.610978500
H	3.378799700	-3.770596800	-2.739325200
C	-1.529939600	-3.256745000	0.966747600
C	-1.691279800	-4.636584200	0.637934900
N	-0.681416700	-2.640212300	0.128389200
C	-0.958820100	-4.837725700	-0.496728500
H	-2.310825900	-5.345271600	1.171634200
C	-0.305850600	-3.597294600	-0.762756500
H	-0.833148200	-5.752217500	-1.061644400
C	0.761886000	-3.514964300	-1.664738600
H	0.876615500	-4.359212500	-2.338347800
N	0.256958200	0.105019000	3.156456600

O	0.435934400	-0.952617700	2.499270300
O	0.030588600	1.123422400	2.453453400
O	0.298183300	0.139168900	4.337949800
Gd	0.160962800	0.024447400	0.344619800

Table S41: Optimised geometry of the $\mathbf{Gd}^{(III)}$**-hex1** complex with the BHLYP/def2-SVP model chemistry.

Atom	X	Y	Z
C	2.8741883	0.9133617	-2.1757505
C	3.2664625	1.7634244	-3.2872607
C	2.4768872	2.8549022	-3.2179151
C	1.6152261	2.6540984	-2.0649830
N	1.7969093	1.4235585	-1.5495372
H	4.0831120	1.5670943	-3.9681171
H	2.5069474	3.7458738	-3.8297151
C	1.0134730	3.6761657	-1.3421131
H	1.1081266	4.6845842	-1.7283543
C	0.5365062	3.5388612	-0.0312276
C	0.5295996	4.6212801	0.9184453
N	0.0851048	2.3978858	0.5431430
C	0.1155334	4.0955611	2.0968925
H	0.8313080	5.6380652	0.7079435
C	-0.2578515	2.7302228	1.8099126
H	-0.0199806	4.5967294	3.0453920
C	-1.2108333	2.0737113	2.5889237
C	3.6956124	-0.0340945	-1.5783545
H	-1.3835467	2.4698439	3.5831950
H	4.6574933	-0.2243866	-2.0405649
C	-2.2263129	1.2885867	2.0426558
C	-3.5804180	1.3882204	2.5353943
N	-2.2065795	0.7228434	0.8128734
C	-4.3781502	1.0302333	1.5001084

H	-3.8688208	1.7748494	3.5031547
C	-3.5035787	0.5859735	0.4459497
H	-5.4584373	1.0362163	1.4538365
C	3.4932385	-0.5551164	-0.2928658
C	4.5729677	-0.9797101	0.5600926
N	2.3045464	-0.6792107	0.3452314
C	4.0148548	-1.3123173	1.7493048
H	5.6185276	-0.9904221	0.2846352
C	2.5867821	-1.2183158	1.5545902
H	4.5033686	-1.6790272	2.6415257
C	1.7117494	-1.9873423	2.3219384
C	-3.9732142	0.0367643	-0.7552175
H	2.0925087	-2.3621901	3.2652300
H	-5.0124065	0.2173105	-1.0053927
C	0.6156013	-2.6575518	1.7784494
C	0.3135583	-4.0147740	2.1696259
N	0.0110773	-2.3531547	0.6061169
C	-0.3400212	-4.5660546	1.1183039
H	0.6477998	-4.4945129	3.0792049
C	-0.5499852	-3.5056145	0.1670131
H	-0.6779065	-5.5863721	1.0000669
C	-3.2947444	-0.9246323	-1.4931734
C	-3.9134236	-1.7998914	-2.4747031
N	-2.1067696	-1.4221921	-1.1004232
C	-3.1245480	-2.8913889	-2.5498462
H	-4.8574892	-1.6177534	-2.9692412
C	-2.0368844	-2.6653765	-1.6126601

H	-3.2825029	-3.7967130	-3.1194532
C	-1.2936763	-3.6719381	-1.0094108
H	-1.4666937	-4.6889190	-1.3424545
Gd	-0.0558425	0.0110611	-0.4000754
N	-0.3600938	-0.0234925	-3.2395521
O	-0.4873899	-0.0384499	-4.4190670
O	-0.9089244	0.8414347	-2.5131471
O	0.3332237	-0.8715266	-2.6254836

Lu-Complexes

Table S42: Optimised geometry of the **Lu$^{(III)}$-pent0** complex with the BHLYP/def2-SVP model chemistry.

Atom	X	Y	Z
C	2.5782298	1.4563860	0.5653999
C	3.7435412	1.2079690	1.3523998
C	3.9311852	-0.1598206	1.3710273
C	2.8788078	-0.7334374	0.5952103
N	2.1563380	0.2769278	0.0515580
H	4.3227883	1.9447565	1.8921437
H	4.6855604	-0.6987233	1.9280090
C	1.5873705	2.4874919	0.5504793
C	1.3913280	3.6780953	1.3135983
N	0.3905736	2.1004550	0.0478912
C	0.0336335	3.9222405	1.3307066
H	2.1541899	4.2371142	1.8382041
C	-0.5828446	2.8764302	0.5787530
H	-0.4741220	4.7091757	1.8716301
C	2.2001271	-1.9907859	0.6071290
C	2.3259996	-3.1773864	1.3941802
N	0.9481524	-1.9502235	0.0937540
C	1.0850314	-3.7765896	1.4122008
H	3.2073793	-3.5010560	1.9308858
C	0.2150950	-2.9487127	0.6362200
H	0.8032341	-4.6617013	1.9660806
C	-1.1931953	-2.7005191	0.6612307

C	-2.2674418	-3.1677880	1.4763394
N	-1.5548474	-1.5082366	0.1262473
C	-3.2239730	-2.1718681	1.4880656
H	-2.2927799	-4.0920958	2.0373779
C	-2.7236207	-1.1076854	0.6794031
H	-4.1418475	-2.1667886	2.0600609
C	-1.8740131	2.2575452	0.6208860
C	-3.0287656	2.4279449	1.4387933
N	-1.9054567	1.0049138	0.0978654
C	-3.6821435	1.2102140	1.4690882
H	-3.2972447	3.3167549	1.9934303
C	-2.9206806	0.3106440	0.6677534
H	-4.5594735	0.9651697	2.0521697
Lu	-0.0004844	-0.0299645	-0.7520923
O	1.5291518	0.0992600	-2.6361221
H	1.5503953	-0.7260513	-3.1213510
O	-0.8140138	-1.3979231	-2.6325335
H	-1.3490060	-0.7748468	-3.1290665
H	2.3213572	0.1301272	-2.0873742
H	-1.4223358	-1.9568185	-2.1405337
O	-1.1624166	1.3146163	-2.5635812
H	-0.6176824	2.0592978	-2.8193397
H	-1.7851548	1.6454812	-1.9041599

Table S43: Optimised geometry of the **Lu$^{(III)}$-porph1212** complex with the BHLYP/def2-SVP model chemistry.

Atom	X	Y	Z
C	2.983715600	1.404764000	0.211024800
C	4.255688000	1.116185900	-0.362170800
C	4.170122800	-0.152287300	-0.855024100
C	2.868771600	-0.626825400	-0.528998300
N	2.152198100	0.369854000	0.111478200
H	5.095129300	1.797070000	-0.396599200
H	4.932777900	-0.726520800	-1.364747000
C	0.452134300	3.271241500	-0.117609200
C	-0.085179600	4.297151000	-0.939584900
N	-0.273845300	2.150306900	-0.190854200
C	-1.172276100	3.752810800	-1.566815200
H	0.324571800	5.292235600	-1.047297300
C	-1.299297500	2.427332200	-1.077586800
H	-1.838843100	4.221558600	-2.279153900
C	1.382968100	-2.664838000	-0.267805900
C	1.328588600	-4.075058100	-0.360123200
N	0.270158200	-2.183245600	0.385539200
C	0.183979600	-4.462727400	0.285837200
H	2.076137500	-4.699991600	-0.830883700
C	-0.454342100	-3.269961800	0.701927800
H	-0.198593200	-5.463971500	0.431257700
C	-2.822806600	0.415542200	-0.841206500
C	-4.118428800	-0.098408500	-1.109392100
N	-2.165385800	-0.395540200	0.064838500

C	-4.265872900	-1.206333500	-0.324824500
H	-4.835578400	0.341792900	-1.789929700
C	-3.030663100	-1.370999900	0.358647000
H	-5.119189000	-1.866062800	-0.245230500
C	2.496198200	-1.942551200	-0.696330700
H	3.239804400	-2.550193400	-1.203636400
C	-2.829215700	-2.530873100	1.212064200
C	-1.755813600	-3.329325600	1.342338600
H	-3.742748700	-2.878747100	1.689700700
H	-1.910308300	-4.239296300	1.918806100
C	1.649737300	3.490288600	0.681472800
C	2.715635300	2.699988000	0.822076200
H	1.714000900	4.481555700	1.125303600
H	3.558021200	3.107268700	1.376538700
C	-2.397159500	1.629530600	-1.356820400
H	-3.090587100	2.070642500	-2.066999700
O	-0.933455900	-0.965110400	3.017757900
H	-0.190428200	-1.495721800	3.337722800
H	-1.673061400	-1.563005100	2.838901700
O	-1.568327700	1.560271700	2.269380700
H	-1.125332800	1.707584500	3.114601300
H	-1.617214500	2.407662500	1.814379600
N	1.788990500	0.317037700	3.303591100
O	1.643611100	-0.798902800	2.764965700
O	1.010499300	1.215765800	2.873464500
O	2.586823200	0.534102000	4.152515600
Lu	-0.017853700	0.101042000	1.095032700

Table S44: Optimised geometry of the **Lu$^{(III)}$-porph1122** complex with the BHLYP/def2-SVP model chemistry.

Atom	X	Y	Z
C	2.856706400	1.630494800	0.187213900
C	3.962518200	1.743050400	-0.687763000
C	4.146056500	0.495555700	-1.216535300
C	3.160180200	-0.351806700	-0.643773900
N	2.339977500	0.399699700	0.192305100
H	4.542158000	2.640244600	-0.855150400
H	4.921160200	0.168352500	-1.897442300
C	0.253791800	3.261959200	0.095928800
C	-0.384128400	4.307925100	-0.631517800
N	-0.352505400	2.091396900	-0.076397000
C	-1.436030400	3.728784900	-1.277318200
H	-0.069526400	5.342472000	-0.647775200
C	-1.436622900	2.353368100	-0.901634600
H	-2.167040000	4.197296300	-1.923666900
C	1.494104900	-3.116308800	0.390137000
C	1.181511900	-4.491943000	0.778262100
N	0.401089200	-2.335751400	0.504672600
C	-0.126307200	-4.534432900	1.058455800
H	1.899742700	-5.300729200	0.781254600
C	-0.639226500	-3.195486300	0.813569400
H	-0.732831500	-5.384812700	1.341278000
C	-2.757253400	0.220782100	-0.699128000
C	-3.982694600	-0.457243800	-0.925626700
N	-1.897652300	-0.575891600	0.017827500

C	-3.867945500	-1.682638300	-0.325805100
H	-4.825465500	-0.058515000	-1.475378100
C	-2.554073900	-1.733239300	0.211628600
H	-4.590192600	-2.487392100	-0.299053900
C	2.740954400	-2.858114600	-0.176062900
C	3.320246800	-1.730899600	-0.735392900
H	3.336421500	-3.757250300	-0.298410000
H	4.193300300	-1.975677100	-1.336359100
C	-1.971758600	-2.920429800	0.751764500
H	-2.651958600	-3.741506200	0.952102800
C	1.375728400	3.481529500	1.001590500
C	2.471474600	2.739209600	1.077398500
H	1.285096100	4.342249000	1.660980000
H	3.222586700	3.008154500	1.818240900
C	-2.498619100	1.512145800	-1.154844100
H	-3.287860600	1.944094600	-1.763165800
O	-1.465003400	1.285564000	2.324713200
H	-1.792370700	0.705654600	3.023011700
H	-2.207907800	1.591390500	1.795058800
O	-0.926353200	-1.203507600	3.008399700
H	-1.420267800	-1.964809800	2.669470800
H	-0.089052800	-1.546835000	3.364850900
N	1.945974200	-0.002924300	3.362881800
O	1.689433400	-1.107963000	2.825234200
O	1.217920400	0.945717900	2.975769500
O	2.800865400	0.140032400	4.170511000
Lu	0.174386900	-0.005854700	1.119295500

Table S45: Optimised geometry of the **Lu$^{(III)}$-sapphyrin** complex with the BHLYP/def2-SVP model chemistry.

Atom	X	Y	Z
C	-0.437539500	-2.140122900	2.703693200
C	-0.580482300	-3.544639600	2.894327900
C	-0.292823900	-4.138354700	1.691697700
C	-0.012340400	-3.077226600	0.794688500
N	-0.138607000	-1.874563000	1.401236000
H	-0.821851700	-4.027617200	3.832396600
H	-0.271156300	-5.194029300	1.457860700
C	-0.362357000	-1.194070200	3.722213700
H	-0.558150300	-1.546295100	4.729488200
C	0.069999800	0.112148500	3.609777700
C	0.360955300	0.906056500	4.767009300
N	0.342545900	0.828635000	2.458245900
C	0.829158000	2.095861400	4.321625200
H	0.225791400	0.576171800	5.788963300
C	0.749070400	2.050537800	2.888973300
H	1.142647100	2.956660700	4.897218300
C	0.871089300	3.213855400	2.128590900
H	1.194168000	4.093626700	2.673833800
C	0.356509400	3.448759900	0.869420700
C	0.056049500	4.796229200	0.447246400
N	-0.091338300	2.543650500	-0.049034600
C	-0.661977600	4.690848400	-0.686699500
H	0.324450200	5.687948700	0.998281400
C	-0.695141700	3.287568300	-1.026403500

H	-1.094691100	5.478900600	-1.288950400
C	-1.130241300	2.894657500	-2.273674600
H	-1.599154600	3.670929200	-2.868333700
C	-0.805686800	1.726235300	-2.968768300
C	-0.885617000	1.665484200	-4.399452800
N	-0.200218000	0.622267400	-2.464956800
C	-0.230051700	0.537424600	-4.771050600
H	-1.337861100	2.420434700	-5.029036200
C	0.170640600	-0.122304800	-3.565111600
H	-0.054078000	0.160401200	-5.770350000
C	0.400446100	-3.099246600	-0.553809200
C	0.738560900	-4.198231300	-1.387179600
N	0.506787500	-1.927331400	-1.212218600
C	1.049980900	-3.657898500	-2.605370900
H	0.736000700	-5.240798100	-1.099855400
C	0.851875800	-2.248612400	-2.492404100
H	1.330550600	-4.178852800	-3.511770200
C	0.747092900	-1.382391600	-3.572932300
H	1.000329100	-1.786367600	-4.547716800
Lu	0.296457400	0.151883000	0.048463700
O	2.259798100	0.768871700	-1.061530800
H	2.974227900	0.134816900	-0.935686000
H	2.062008500	0.874054900	-2.000759000
O	2.404808200	-0.669486100	1.095559400
H	2.109378300	-1.563346900	1.316879300
H	2.335297300	-0.171658100	1.922829400
O	-1.966909300	-0.218067700	-0.288336400

H	-2.065441400	-0.198334700	-1.251687300
H	-2.204840600	-1.096523100	0.033174400

Table S46: Optimised geometry of the $\mathbf{Lu}^{(III)}$**-porph2** complex with the BHLYP/def2-SVP model chemistry.

Atom	X	Y	Z
C	2.741503900	0.978259900	-0.584896400
C	4.177610900	0.813394600	-0.565734500
C	4.457495800	-0.011367300	0.460027000
C	3.187860800	-0.474509200	0.965467700
N	2.161067100	0.160745900	0.346982400
H	4.864200400	1.326910900	-1.225256600
H	5.421463900	-0.347537300	0.816843300
C	-0.072581900	2.942694100	-0.691895200
C	-0.727733600	4.225472600	-0.736719300
N	-0.782241700	2.079992500	0.107540400
C	-1.790517900	4.151665700	0.089573800
H	-0.380504200	5.072578700	-1.312937100
C	-1.857584300	2.783254600	0.529621100
H	-2.521775900	4.913772400	0.322223600
C	1.310876800	-3.053811600	1.200720400
C	1.213701600	-4.439202900	0.885424000
N	0.564679700	-2.309161700	0.316436000
C	0.533834500	-4.510554000	-0.296858100
H	1.704689100	-5.238986700	1.424629100
C	0.116734400	-3.185446400	-0.603243700
H	0.314123500	-5.386967400	-0.892072900
C	-3.444483100	-0.019722900	0.313135800
C	-4.536152500	-0.678402100	-0.316695500
N	-2.247731700	-0.491814000	-0.172106400

C	-3.993947800	-1.446452500	-1.303508800
H	-5.581734200	-0.496430300	-0.105416700
C	-2.581642100	-1.349514300	-1.158451700
H	-4.504372500	-2.055785400	-2.037413100
C	2.368609900	-2.621652900	2.020119500
C	3.195817600	-1.534853500	1.892929600
H	2.719010200	-3.381819100	2.713641700
H	4.114280700	-1.590318300	2.469774200
C	-1.768514900	-2.179563700	-1.996395000
C	-0.681801400	-2.948611900	-1.766656200
H	-2.247597900	-2.368472400	-2.954379000
H	-0.452878000	-3.649226000	-2.566833900
C	1.107639400	2.802414200	-1.406825500
C	2.232556800	1.980772700	-1.397205700
H	1.240418500	3.638438500	-2.087859800
H	2.984570300	2.328054200	-2.099469000
C	-3.726885900	1.152680400	1.037249200
C	-3.054419500	2.348850300	1.149460700
H	-4.744448400	1.176565100	1.419750000
H	-3.634013000	3.158460100	1.583521700
N	1.418663800	0.846778900	3.302974100
O	0.972130500	-0.311383100	3.082779500
O	0.943384200	1.739035800	2.570457400
O	-1.460086000	0.784879100	2.918375800
H	-0.900561500	1.548561400	3.128816000
H	-2.333655500	1.130586200	2.687100100
O	2.226506700	1.055178900	4.146883800

O	-1.219031300	-1.742163700	2.420774100
H	-0.758122300	-2.588933400	2.391101000
H	-1.132323100	-1.375233100	3.309184300
Lu	-0.156848800	-0.087661600	1.046032800

Table S47: Optimised geometry of the **Lu$^{(III)}$-amethyrin** complex with the BHLYP/def2-SVP model chemistry.

Atom	X	Y	Z
C	3.746076800	-1.174605500	0.501073400
C	4.426592000	-2.438440400	0.534364700
C	3.469280100	-3.401358400	0.489116900
C	2.224327300	-2.701087500	0.429713000
N	2.399801500	-1.369360200	0.439628100
H	5.499155100	-2.564622900	0.585973600
H	3.596863400	-4.473861200	0.495482800
C	3.713274100	1.273881000	0.503920600
C	4.359572900	2.555579700	0.538884300
N	2.362256100	1.432455800	0.442964700
C	3.376720100	3.492400300	0.494690300
H	5.428354300	2.710522000	0.590616700
C	2.151023100	2.758882800	0.434283500
H	3.475401700	4.567934200	0.502225800
C	0.839291100	3.241929700	0.377980700
C	0.394722600	4.596819600	0.365554400
N	-0.192597500	2.372386300	0.345825000
C	-0.963940500	4.535173100	0.331308300
H	1.024859700	5.473913100	0.384172400
C	-1.309690400	3.146347900	0.329891400
H	-1.664951900	5.356713600	0.319214100
C	-2.611422000	2.635590200	0.356010400
C	-3.861792700	3.395704500	0.324092200
N	-2.827823800	1.335450300	0.423962700

C	-4.836385800	2.467983100	0.370336000
H	-3.964581800	4.469680000	0.267084400
H	-5.907102600	2.620540400	0.359644000
C	0.926026800	-3.219218300	0.374048900
C	0.518063600	-4.585660800	0.360487700
N	-0.128857300	-2.377769400	0.343882700
C	-0.841750600	-4.560552300	0.327445100
H	1.171592300	-5.445511700	0.377513000
H	-1.520415800	-5.400648000	0.314955300
C	-1.224775800	-3.181456800	0.327844500
C	-4.157994300	1.171178500	0.425733300
C	-4.768373200	-2.598101700	0.369311800
C	-3.769230700	-3.499317300	0.322592000
C	-4.125008000	-1.283596100	0.425445400
H	-5.834615800	-2.779297800	0.358420500
C	-2.539708800	-2.705941200	0.354946500
N	-2.790985800	-1.412062500	0.423771900
H	-3.843150500	-4.575634900	0.264903200
C	4.372296000	0.058264900	0.529526300
C	-4.793727900	-0.064923700	0.443270800
H	5.454657200	0.072678100	0.577064800
H	-5.878127200	-0.079492800	0.438729100
Lu	0.469807400	0.006199500	0.003558900
N	0.156624000	0.006253800	-2.686851400
O	-0.822548700	-0.014280600	-1.902033400
O	1.277690800	0.025554200	-2.090878000
O	0.058035900	0.007702200	-3.859735200

Table S48: Optimised geometry of the **Lu$^{(III)}$-isoamethyrin** complex with the BHLYP/def2-SVP model chemistry.

Atom	X	Y	Z
C	3.792340600	-0.346075600	-0.107282300
C	5.082108200	-1.011870800	-0.137193700
C	4.823760200	-2.294293100	0.206053300
C	3.383645000	-2.366543900	0.416626900
N	2.810977000	-1.186603700	0.213824400
H	6.035513900	-0.563696300	-0.378464300
H	5.522689000	-3.114116000	0.301993600
C	3.480937700	1.006195400	-0.214520200
C	4.395300300	2.125124300	-0.294239500
N	2.219210700	1.429154000	-0.019665400
C	3.653354400	3.234314000	-0.067142000
H	5.460796100	2.057823200	-0.461135200
C	2.304031600	2.763088000	0.126433900
H	3.982061700	4.262477500	-0.015960300
C	1.144754600	3.428825800	0.501777200
C	0.834938100	4.798662900	0.760753500
N	0.036553700	2.665216700	0.614426300
C	-0.510153600	4.841916300	1.004323000
H	1.531320900	5.624987500	0.746320800
C	-0.974566900	3.495894500	0.876542500
H	-1.116106700	5.709769300	1.221646700
C	-2.260373900	2.935175100	0.798116300
C	-3.544904200	3.555477700	0.845319600
N	-2.336549400	1.636385100	0.466688900

C	-4.426959300	2.584817400	0.483561600
H	-3.746032000	4.587526400	1.093900500
H	-5.500911800	2.660492500	0.382738800
C	1.268972500	-3.584607300	0.779747500
C	0.605935800	-4.871881700	0.876090000
N	0.354156200	-2.594828800	0.538653200
C	-0.695796600	-4.650943600	0.611430900
H	1.101729300	-5.811685700	1.076606800
H	-1.500062200	-5.369092300	0.562213300
C	-0.801446300	-3.220031500	0.404143500
C	-3.654676500	1.398149700	0.250112900
C	-4.148258800	-2.256124900	-0.412238200
C	-3.230015000	-3.230516100	-0.196949500
C	-3.469831700	-1.010648600	-0.229442700
H	-5.200631600	-2.360532000	-0.636419000
C	-2.002697300	-2.554380600	0.077894400
N	-2.136071100	-1.216182000	0.026142400
H	-3.373262900	-4.299693500	-0.221888100
C	-4.169186200	0.180330100	-0.145651800
H	-5.234442700	0.130333800	-0.339306100
Lu	-0.213548800	0.389116500	-0.043924300
C	2.645510100	-3.501687700	0.756414000
H	3.189814000	-4.422441500	0.936078500
O	-0.074036000	0.325012900	-3.921601200
O	-0.658420300	1.246127600	-2.070706500
O	0.386528100	-0.584274900	-2.031876000
N	-0.109508600	0.323197200	-2.745171600

Table S49: Optimised geometry of the **Lu$^{(III)}$-pent1** complex with the BHLYP/def2-SVP model chemistry.

Atom	X	Y	Z
C	1.2034492	-1.3428876	-3.9115582
C	0.8786700	-2.6140559	-3.3266179
C	-0.0585393	-3.1702575	-4.1341587
C	-0.1931277	-2.2860679	-5.2574813
N	0.5576760	-1.1671326	-5.0837131
H	1.2767531	-2.9945121	-2.3962083
H	-0.5686827	-4.1166563	-4.0195276
C	1.9422814	-0.3757018	-3.2223209
H	2.5044389	-0.7259965	-2.3641701
C	1.7740668	0.9884200	-3.3234179
C	2.0818300	1.8357606	-2.1934070
N	0.9948660	1.6526998	-4.2281270
C	1.3454710	2.9528710	-2.3552542
H	2.7058115	1.5522949	-1.3571356
C	0.6845530	2.8204615	-3.6320505
H	1.2471129	3.8006072	-1.6916508
C	-0.1642097	3.8126459	-4.1215610
H	-0.4662745	4.5773867	-3.4162262
C	-0.5138146	4.0152651	-5.4366745
C	-1.1681814	5.2328010	-5.8700526
N	-0.0717958	3.2973140	-6.5042120
C	-1.0120152	5.2887576	-7.2041371
H	-1.6254056	5.9563282	-5.2096605
C	-0.2846770	4.0939321	-7.5801906

H	-1.3149279	6.0675501	-7.8899931
C	0.2766504	3.9494506	-8.8326814
C	-0.7698999	-2.7029575	-6.4524171
H	-1.3588599	-3.6122628	-6.4186803
H	0.1513761	4.7824335	-9.5138593
C	1.0423498	2.8885355	-9.2995469
C	1.7655184	2.9724814	-10.5491054
N	1.1435335	1.6548773	-8.7580674
C	2.2858021	1.7514635	-10.7714172
H	1.8335851	3.8589025	-11.1642412
C	1.8064551	0.9106051	-9.6948809
H	2.8628691	1.4053026	-11.6177120
C	-0.3855008	-2.2515579	-7.7054485
C	-0.4171224	-3.1122558	-8.8580923
N	0.3850488	-1.1562192	-7.9438485
C	0.4607209	-2.5825456	-9.7432198
H	-0.9766714	-4.0342479	-8.9344956
C	0.9187205	-1.3476797	-9.1637264
H	0.7533485	-2.9644756	-10.7114291
C	1.7481432	-0.4575116	-9.8565568
H	2.2293519	-0.8509743	-10.7446419
Lu	0.3309303	0.8851591	-6.5140180
N	-2.4293292	0.9300513	-6.6209175
O	-1.7230000	1.0129505	-7.6532394
O	-1.7694057	0.7667037	-5.5578882
O	-3.6106201	0.9975811	-6.6356020
O	2.6395521	0.3265354	-6.5622188

H	2.6787636	-0.5604063	-6.1956825
H	2.9835923	0.2725733	-7.4555964

Table S50: Optimised geometry of the **Lu$^{(III)}$-rosarin** complex with the BHLYP/def2-SVP model chemistry.

Atom	X	Y	Z
C	1.978787300	2.693912000	-0.828247600
C	2.281742600	4.075252500	-0.957724100
C	1.303629000	4.729280100	-0.251461300
C	0.416537400	3.729998000	0.231548100
N	0.845481700	2.486497200	-0.161449600
H	3.143426200	4.500214100	-1.453521900
H	1.200102900	5.794760000	-0.089119200
C	-0.732431600	3.968079800	0.975976900
H	-0.889741300	4.986220600	1.320474600
C	-1.767693500	3.071338500	1.174295500
C	-3.104810000	3.481840500	1.466121100
N	-1.778555900	1.808560600	0.610624700
C	-3.926093700	2.503101100	0.978577300
H	-3.387674400	4.428916400	1.908142300
C	-3.057042700	1.510123800	0.443765400
H	-5.006938100	2.477801600	0.970932100
C	-3.398406500	0.324993200	-0.294500000
C	-4.643121900	-0.037297900	-0.861641800
N	-2.454322800	-0.589322200	-0.509355200
C	-4.435784400	-1.266439600	-1.442086900
H	-5.556571700	0.540266600	-0.827715100
C	-3.087917500	-1.618351900	-1.164406400
H	-5.159744200	-1.895401100	-1.944371300
C	2.892753200	1.588782800	-0.978436400

C	4.246897400	1.650988900	-1.404680100
N	2.616806800	0.462719100	-0.324834800
C	4.842465900	0.534483000	-0.882752000
H	4.706983600	2.457603200	-1.958528800
C	3.829539700	-0.178709600	-0.182395000
H	5.885547000	0.249142300	-0.935596100
C	4.069260700	-1.169644200	0.750479000
C	-2.638106800	-2.933855400	-1.119458600
H	5.106939100	-1.447166600	0.911456200
H	-3.266747200	-3.686156500	-1.586574500
C	3.115582200	-1.874215300	1.473453400
C	3.395634600	-3.018133700	2.267095400
N	1.772355500	-1.824599300	1.177619900
C	2.216686400	-3.712528300	2.385238600
H	4.373756700	-3.301944200	2.634645600
C	1.265786800	-2.963631700	1.649995100
H	2.048056100	-4.664959200	2.868479600
C	-1.590356000	-3.391191000	-0.345715800
C	-1.445445000	-4.743196000	0.078186200
N	-0.657409100	-2.597675500	0.288068100
C	-0.447614300	-4.756015000	1.014587500
H	-2.050307000	-5.573767400	-0.262790900
C	0.025439300	-3.417268800	1.078647000
H	-0.054599500	-5.600596700	1.563015400
O	-0.242404900	0.455127700	-2.129146300
H	-0.010014200	1.387891000	-2.238507300
H	-1.197401500	0.359008700	-2.256718100

O	0.106684500	0.571631600	2.492587000
H	0.874938600	0.477080100	3.065390800
H	-0.315587500	1.429687100	2.638082000
Gd	0.108134500	-0.102636800	0.195112700

Table S51: Optimised geometry of the **Lu$^{(III)}$-rubyrin** complex with the BHLYP/def2-SVP model chemistry.

Atom	X	Y	Z
C	1.832013000	3.276839000	0.739439100
C	1.984097200	4.625482300	0.295296400
C	1.242102700	4.728638200	-0.847090900
C	0.594448900	3.467249000	-1.004244100
N	0.977404400	2.591899500	-0.036730900
H	2.605483700	5.378544700	0.761926900
H	1.109989300	5.592003700	-1.486139700
C	-0.472762800	3.284359400	-1.890762200
H	-0.609739300	4.056610000	-2.642010600
C	-1.511005000	2.387054200	-1.727995700
C	-2.846367100	2.700863700	-2.131027500
N	-1.536685200	1.438805800	-0.734524000
C	-3.678028700	2.024656100	-1.277271600
H	-3.118960500	3.414955800	-2.897323100
C	-2.819579200	1.273569600	-0.434062300
H	-4.757819000	2.052768400	-1.232659000
C	2.737832900	2.721991200	1.694602200
H	3.315589200	3.430218900	2.280338000
C	-3.125959600	0.390564700	0.664502500
C	-4.330586700	0.191555300	1.424819900
N	-2.171469200	-0.424690900	1.031087500
C	-4.057606100	-0.833557700	2.276807400
H	-5.254111300	0.743249000	1.317816500
C	-2.723476000	-1.285727200	1.951625100

H	-4.726499700	-1.304923900	2.985019600
C	3.145895400	1.422085600	1.671789600
C	4.500286700	0.987976500	1.930730900
N	2.534448800	0.509279000	0.843330400
C	4.716981700	-0.085975000	1.123838800
H	5.214927300	1.499424300	2.562291000
C	3.463709100	-0.327819800	0.461009700
H	5.632468600	-0.644286400	0.986445100
C	-2.313010500	-2.583098800	2.022222500
H	-2.848218700	-3.254528700	2.686462200
C	3.085050800	-1.273666600	-0.559849700
C	3.885646900	-2.075258000	-1.413273500
N	1.784975300	-1.453767600	-0.762093100
C	2.999179800	-2.799206700	-2.166929400
H	4.966023800	-2.103054900	-1.440500900
C	1.693716000	-2.459174700	-1.693616500
H	3.220489500	-3.558030100	-2.906284400
C	-1.473566100	-3.195079900	1.041499900
C	-1.653272300	-4.567154500	0.688610900
N	-0.674699800	-2.558944000	0.170354600
C	-0.989945300	-4.739064800	-0.493161500
H	-2.240656900	-5.289956900	1.239521600
C	-0.356406700	-3.490559200	-0.767590400
H	-0.900011500	-5.638798300	-1.087825400
C	0.648419300	-3.362386200	-1.732885300
H	0.736254600	-4.177764600	-2.444867500
Lu	0.164365500	0.026836100	0.413338200

N	0.257432400	0.103943400	3.146829200
O	0.452574600	-0.947617200	2.484613500
O	0.015623600	1.116881300	2.441134900
O	0.297661700	0.137382900	4.328090700

Table S52: Optimised geometry of the $\mathbf{Lu^{(III)}}$**-hex1** complex with the BHLYP/def2-SVP model chemistry.

Atom	X	Y	Z
C	2.8281326	0.7727812	-2.2071189
C	3.2534514	1.6178195	-3.3096840
C	2.4658696	2.7140507	-3.2611593
C	1.5819288	2.5187390	-2.1263512
N	1.7454739	1.2773862	-1.6127607
H	4.0880140	1.4186022	-3.9675440
H	2.5178996	3.6061361	-3.8699042
C	1.0169231	3.5337813	-1.3848501
H	1.1092519	4.5480981	-1.7557879
C	0.5744685	3.3723755	-0.0613950
C	0.6068738	4.4311843	0.9109537
N	0.1357531	2.2235283	0.4985519
C	0.2252709	3.8816073	2.0915545
H	0.9075848	5.4508369	0.7134265
C	-0.1652931	2.5303280	1.7854837
H	0.1209131	4.3632579	3.0539634
C	-1.1224606	1.8704219	2.5561362
C	3.6419652	-0.1718791	-1.5726685
H	-1.2804734	2.2368829	3.5642612
H	4.6126321	-0.3752560	-2.0092177
C	-2.1501920	1.1221695	1.9861819
C	-3.5035596	1.2310615	2.4654749
N	-2.1268032	0.6054557	0.7310076
C	-4.2956614	0.9276156	1.4064862

H	-3.7972945	1.5871218	3.4432856
C	-3.4173221	0.5041343	0.3487299
H	-5.3749768	0.9560016	1.3480533
C	3.4167435	-0.6146967	-0.2809744
C	4.4853337	-1.0192106	0.6091009
N	2.2222804	-0.6460408	0.3755950
C	3.9250039	-1.2184103	1.8198617
H	5.5278549	-1.0942831	0.3318045
C	2.4940342	-1.0936039	1.6168260
H	4.4024441	-1.5176016	2.7425999
C	1.5945771	-1.7573384	2.4534089
C	-3.8832398	-0.0318432	-0.8645933
H	1.9576504	-2.0607754	3.4281754
H	-4.9155530	0.1642481	-1.1305237
C	0.5288179	-2.4803376	1.9184824
C	0.2330594	-3.8191350	2.3939673
N	-0.0375864	-2.2625848	0.7138844
C	-0.3495100	-4.4547786	1.3572349
H	0.5337834	-4.2239915	3.3503049
C	-0.5522809	-3.4620607	0.3217402
H	-0.6537659	-5.4903133	1.2903233
C	-3.2050023	-1.0031559	-1.5679088
C	-3.8223373	-1.9366645	-2.4937795
N	-2.0173451	-1.4882349	-1.1357526
C	-3.0478210	-3.0424674	-2.4751013
H	-4.7609001	-1.7821450	-3.0078761
C	-1.9662692	-2.7561448	-1.5471917

H	-3.2145193	-3.9881704	-2.9719353
C	-1.2418120	-3.7272020	-0.8489844
H	-1.4209130	-4.7662310	-1.0988629
Lu	-0.0441219	-0.0692370	-0.4353141
O	0.4240923	-1.0654828	-2.4994704
N	-0.2510918	-0.2580976	-3.1845570
O	-0.3377574	-0.3377933	-4.3648852
O	-0.8268973	0.6405971	-2.5229291

References

(1) Shannon, R. D. Revised effective ionic radii and systematic studies of interatomic distances in halides and chalcogenides. *Acta. Crystallogr. A* **1976**, *32*, 751–767.
